# Infection of a β-galactosidase-deficient mouse strain with Theiler’s murine encephalomyelitis virus reveals limited immunological dysregulations in this lysosomal storage disease

**DOI:** 10.3389/fimmu.2025.1467207

**Published:** 2025-04-09

**Authors:** Rouven Wannemacher, Felix Stegmann, Deborah Eikelberg, Melanie Bühler, Dandan Li, Sayali Kalidas Kohale, Thanaporn Asawapattanakul, Tim Ebbecke, Marie-Kristin Raulf, Wolfgang Baumgärtner, Bernd Lepenies, Ingo Gerhauser

**Affiliations:** ^1^ Department of Pathology, University of Veterinary Medicine Hannover, Foundation, Hannover, Germany; ^2^ Center for Systems Neuroscience Hannover, Hannover, Germany; ^3^ Institute for Immunology, University of Veterinary Medicine Hannover, Foundation, Hannover, Germany; ^4^ Research Center for Emerging Infections and Zoonoses (RIZ), University of Veterinary Medicine Hannover, Foundation, Hannover, Germany; ^5^ Institute for Parasitology, University of Veterinary Medicine Hannover, Foundation, Hannover, Germany; ^6^ Chair of Biochemistry and Chemistry, Veterinary Faculty, Ludwig-Maximilians-Universität München, Munich, Germany

**Keywords:** β-galactosidase deficiency, brain, GM1 gangliosidosis, microglia activation, T cell activation, Theiler’s murine encephalomyelitis virus

## Abstract

**Introduction:**

A hallmark of many lysosomal storage diseases (LSD) is the alteration of immune responses, often starting before the onset of clinical disease. The present study aimed to investigate how G_M1_ gangliosidosis impacted the course of an acute central nervous system (CNS) virus infection before the clinical onset of LSD.

**Methods:**

For this purpose, *Glb1* ^-/-^ and wildtype control mice (both C57BL/6 background) were intracerebrally infected with the BeAn strain of Theiler’s murine encephalomyelitis virus (TMEV) at the age of 5 weeks and sacrificed 4, 7, 14 and 98 days post infection, respectively. Histology, immunohistochemistry, and flow cytometry was used to assess viral load and immune cell activation and infiltration.

**Results:**

Both wildtype and *Glb1* ^-/-^ mice were able to clear the virus from the CNS and did not develop any clinical symptoms of TMEV-associated disease, thus indicating no overt alteration in susceptibility to TMEV infection. However, in the early phase post infection, *Glb1* ^-/-^ mice displayed a slightly delayed T cell response as well as an increase in the number and activation of CNS microglia.

**Discussion:**

These results suggest that already in the early stage of disease (before clinical onset) G_M1_ gangliosidosis causes an impaired T cell response and microglial hyperreactivity.

## 1 Introduction

G_M1_ gangliosidosis is a lysosomal storage disease (LSD) caused by mutations in the β-galactosidase (*Glb1*) gene impeding its enzyme activity (1). Aside from humans it has been described in cats (2–4), dogs (5–8), sheep (9–12), cattle (13, 14), emus (15, 16) and American Black Bears (17). Additionally, it has been induced in several mouse models (18–22). *Glb1* deficiency leads to lysosomal accumulation of the ganglioside G_M1_, a sialic acid-containing glycosphingolipid, and other glycoproteins mainly in the central nervous system (CNS) (23, 24). In humans, three distinct clinical manifestations are distinguished: the infantile form is characterised by an early onset (birth to 6 months of age) (24). Symptoms include CNS degeneration, hepatosplenomegaly and facial and skeletal abnormalities (24). The late infantile or juvenile form becomes clinically apparent between 7 months and 3 years after birth and is characterized by progressive motor problems and seizures (24). The adult form is defined by first symptoms appearing between the age of 3 and 30 years and results in cerebellar dysfunction, dystonia and mild vertebral abnormalities (23, 24).

LSDs have been shown to have a variety of effects on immune homeostasis and induced immune responses (25). Gaucher disease, mucopolysaccharidosis VII and α-mannosidosis cause a predisposition towards immunosuppression, while G_M2_ gangliosidosis (Tay-Sachs disease and Sandhoff disease), globoid cell leukodystrophy, Niemann-Pick disease type C (NPC), Fabry disease, and juvenile neuronal ceroid lipofuscinosis predispose towards immune hyperreactivity (25, 26). In NPC, sphingolipid accumulation results in alterations in mTORC1, VEGF/SphK, c-Abl/HDAC2, insulin, and interferon (IFN) signaling pathways and calcium-homeostasis of neurons and glial cells (26). Through yet unknown mechanisms, neuronal loss in NPC leads to early activation of glial cells contributing to CNS disease (26). Mouse models of G_M1_ and G_M2_ gangliosidoses also demonstrated a progressive increase in microglial activation/expansion and T cell infiltration into the CNS with alterations in blood–brain barrier permeability as well as elevated major histocompatibility complex (MHC) class II expression and cytokine levels (TNF, IL-1β and TGF-β1) (27). Furthermore, autoantibodies directed against gangliosides have been demonstrated to contribute to disease in human G_M2_ gangliosidosis and its corresponding mouse model (β-hexosaminidase (*Hexb*)^-/-^ mice) (26, 28). Anti-ganglioside antibodies were also observed in patients suffering from Guillain-Barré syndrome and amyotrophic lateral sclerosis further implicating an autoimmune response against undegraded gangliosides in the progression of neurodegenerative diseases (26, 28). In *Glb1^-/-^* C57BL/6 mice, ganglioside accumulation occurs mainly in CNS neurons leading to progressive neurologic disorder starting at an age between 3.5 to 4 months, while skeletal malformations and organomegaly are not observed (21). Histologically, neurons in the brain stem, cerebellum, cerebral cortex and thalamus display progressive vacuolation (21). Interestingly, axonopathy and reduction of membrane resistance were key features in this new murine model of human G_M1_ gangliosidosis (21).

Theiler’s murine encephalomyelitis virus (TMEV) is a single stranded RNA virus of the genus *Cardiovirus* of the *Picornaviridae* family (29–31). Two subgroups of the virus are distinguished: the highly virulent GDVII subgroup, and the less virulent Theiler’s Original (TO) subgroup (32, 33). The main representatives of the TO subgroup are the BeAn- and Daniel’s (DA)-strains (32, 34, 35). Mouse strains differ in their susceptibility to TMEV-induced demyelinating disease (TMEV-IDD) (32). Resistant strains like C57BL/6 are able to clear the virus from the CNS (36–40), whereas susceptible strains (especially SJL mice) (40–42) develop a biphasic disease (32, 36). The acute phase is characterized by polioencephalomyelitis with virus persistence in neurons, sometimes leading to severe neurological disorders (32). The virus is suspected to spread via axonal as well as haematogenous transport in susceptible animals (43). The chronic phase of disease is characterized by predominantly T cell-mediated demyelinating leukomyelitis with virus persistence mainly in microglia, oligodendrocytes and astrocytes (32). Clinically, animals display progressive lameness of the hind limbs leading up to paraplegia and incontinence (34–36, 44–46). As TMEV-IDD causes progressive demyelinating disease, it represents a widely accepted animal model for the chronic progressive form of human Multiple Sclerosis (39, 47–49).

While it is known that LSDs may affect the immune response, currently there is a knowledge gap on how progressive lysosomal storage in *Glb1* ^-/-^ C57BL/6 mice might affect axonal spread of TMEV and antiviral responses. Therefore, the aim of the present study was to determine how *Glb1* deficiency affected the immune response to TMEV infection. To this end, *Glb1^-/-^* and wildtype (*Glb1^+/+^*) control mice with a C57BL/6 background were infected intracerebrally with the BeAn strain of TMEV and sacrificed at 4, 7, 14, and 98 days post infection (dpi). Both *Glb1^+/+^* and *Glb1^-/-^* mice were able to eliminate the virus from their CNS without exhibiting any clinical symptoms of TMEV-related illness. Nonetheless, during the early post-infection phase, *Glb1^-/-^* mice exhibited a slightly delayed T cell response and an elevated activation state of CNS microglia compared to C57BL/6 wildtype mice. These findings suggest that prior to the onset of clinical symptoms, G_M1_ gangliosidosis induces enhanced microglial reactivity and restricted T cell responses in TMEV infection. Consequently, innate and adaptive immune reactions to neurotropic virus infections are likely to be disturbed in several LSD, which could result in a higher susceptibility of affected patients to severe CNS disease. Moreover, a delay in viral elimination and increased microglia activation status might predispose to long-term consequences of viral encephalitis including neurocognitive impairment, neuropsychiatric disorders, and epilepsy.

## 2 Material and methods

### 2.1 Animals

Homozygous *Glb1* ^em1/RASE-/-^Tg(LacZ)C57BL/6 (*Glb1^-/-^*) mice and C57BL/6 wildtype (*Glb1^+/+^*) littermates were generated by breeding of heterozygous animals from a previously established population (21). Animals were housed in individually ventilated cages (Tecniplast Deutschland GmbH, Hohenpeißenberg, Germany) with 12 h light and 12 h darkness at 22 - 24°C and 50 - 60% humidity. Food for maintenance and breeding (ssniff Spezialdiäten GmbH, Soest, Germany) as well as water were provided *ad libitum*. Enrichment of the cages included mouse houses (Tecniplast Deutschland GmbH) and nesting material (ssniff Spezialdiäten GmbH).

DNA was extracted from ear notch tissue taken from up to three-week-old mice. Standard genotyping of the mice was performed using PCR with the forward primer *Glb1* Primer 0 fwd (5’-CTG TTG GCT TGA GAC CAG TGT AGT C-3’) binding in intron 14 and the reverse primer *Glb1* Primer 0 rev (5’-GAT GCA TAC CTT GGA CCA CCC AG-3’) binding in exon 15 of the *Glb1* gene (21).

The study was approved by the Local Institutional Animal Care and Research Advisory committee and permitted by the appropriate authority (LAVES, Oldenburg, Germany, permission number: 33.8-42502-04-19/3204).

### 2.2 Experimental design

C57BL/6 *Glb1^+/+^* and homozygous *Glb1^-/-^* mice were intracerebrally infected with 1x10^5^ plaque forming units (PFU) of TMEV-BeAn at 5 weeks of age under general anaesthesia. An equal number of control *Glb1^-/-^* and *Glb1^+/+^* mice was intracerebrally injected with virus-free cell solution (mock/placebo). General anaesthesia was performed via intraperitoneal injection of Medetomidin (Domitor^®^, Pfizer 1.0 mg/ml) and Ketamin (Ketamin 10%, WDT, 100 mg/ml) diluted in NaCl at a dosage of 0.5 mg/kg Medetomidin and 100 mg/kg Ketamin. Starting at 0 dpi, mice were examined weekly for general appearance and posture, behaviour and activity as well as gait. Further examination included measuring of the body weight, parachute test (50), grid-walking test (50), hang test (51) and RotaRod performance test (RotaRod Treadmill, TSE Technical & Scientific Equipment, Bad Homburg, Germany) as described before (52). Mice were placed on a rotating rod, with the rotation speed steadily accelerating from 5 rounds per minute (rpm) to 30 rpm over the course of 300 seconds. Each mouse was tested three times, and the highest rod turn rate (rounds per minute (rpm)), that was tolerated at each trial before mice fell off, was recorded and averaged. All clinical and neurological tests performed are described in detail in **Supplementary Table 1**. For euthanasia, mice were first put in general anaesthesia as described above and then humanely killed via a second, undiluted intraperitoneal injection of Medetomidin (Domitor^®^, Pfizer 1.0 mg/ml) and Ketamin (Ketamin 10%, WDT, 100 mg/ml) at a dosage of 1 mg/kg Medetomidin and 200 mg/kg Ketamin. After death, mice were perfused with phosphate buffered saline (PBS). Following euthanasia at 4, 7, 14 and 98 dpi, respectively, tissue samples were taken from brain, spinal cord and spleen. Samples were fixed overnight in 10% neutral buffered formalin, decalcified with EDTA (only spinal cord with adjacent vertebrae) and embedded in paraffin. Additional organ samples were embedded in OCT medium, frozen in liquid nitrogen and stored at -80°C. Tissue samples from both brain (front left quarter of cerebrum) and spleen were taken and stored in Iscove’s Modified Dulbecco’s Medium (IMDM) supplemented with 10% FCS, 2 mM L-glutamine, and 100 U/mL penicillin/streptomycin until further flow cytometry analysis. All analyses performed in the study were performed once (number of repeat experiments = 1).

### 2.3 Histology and immunohistochemistry

Histology and immunohistochemistry were performed on paraffin sections applying the avidin-biotin-peroxidase complex method (Vector Laboratories Inc., Burlingame, CA, USA) with the chromogen 3,3’-diaminobenzidine-tetrahydrochloride (DAB) as previously described (53–57). Primary antibodies directed against glial fibrillary acidic protein (GFAP), amyloid precursor protein (APP), TMEV, CD3, Iba1 and CD107b (Mac3) were used to detect astrocytes, axonal lesions, virus, T cells, microglia/macrophages and activated microglia/macrophages cells, respectively (**Table 1**).

**Table 1:** Specifications of the antibodies used for immunohistochemistry.

Antibody	Target	Producer	Dilution
Rabbit anti-TMEV, polyclonal	TMEV	Kummerfeld et al., 2009 (81)	1:2000
Mouse anti-amyloid precursor protein (APP) A4, monoclonal (clone 22C11)	β Amyloid precursor protein (APP)	Merck KGaA Cat.: MAB348	1:2000
Rabbit anti-Iba1, polyclonal	Microglia/macrophages	Wako Cat.: 019-19741	1:2000
Rat anti-mouse CD107b, monoclonal	Activated microglia/macrophages	Bio-Rad Cat.: MCA2293	1:800
Rabbit anti-cow glial fibrillary acidic protein (GFAP), polyclonal	Astrocytes	DAKO Cat.: Z-0334	1:1000
Rabbit anti-human CD3, polyclonal	T-lymphocytes	DAKO Cat.: A-0452	1:2000

Haematoxylin and Eosin (H&E) stains were evaluated for perivascular cuffing, i.e. layered perivascular accumulation of infiltrating leukocytes using a semiquantitative scoring system going from 0 to 3 (0 – normal, 1 = single perivascular infiltrates, 2 = 2-3 layers of perivascular infiltrates, 3 = >3 layers of perivascular infiltrates). Cortex, hippocampus, diencephalon/mesencephalon, cerebellum and *medulla oblongata* were scored separately, and the scores were combined into an average brain score.

Immunostainings were analysed by counting the number of APP positive spheroids, CD3 positive T cells, and TMEV positive cells in cortex, hippocampus, diencephalon/mesencephalon, cerebellum and medulla oblongata (regions of interest (ROI)) in the brain, resulting in a cumulative, total number of positive cells per brain section. Stainings for GFAP, Iba1 and CD107b were evaluated by measuring the relative positive brain area through the use of the QuPath software (QuPath 0.5) (58). Perivascular infiltration of Iba1-positive macrophages at 4 and 7 dpi was investigated semiquantitatively analogous to the scoring system described above for perivascular cuffing on H&E sections.

### 2.4 Real-time quantitative polymerase chain reaction

RNA was isolated from frozen brain tissue using the RNeasy^®^ Lipid Tissue Mini Kit (Qiagen, Hilden, Germany) following the manufacturers’ instructions. The Omniscript RT Kit (Qiagen), Random Primers (Promega, Mannheim, Germany) and RNase Out (Invitrogen, Darmstadt, Germany) were used for the generation of cDNA. RT-qPCR was performed using the AriaMx Real-time PCR System (Agilent Technologies Deutschland GmbH) and the Brilliant III Ultra-Fast SYBR^®^QPCR Master Mix as described (59). The following primers were used: TMEV *forward* (GAC TAA TCA GAG GAA CGT CAG C), TMEV *reverse* (GTG AAG AGC GGC AAG TGA GA), IFN-γ *forward* (CAC GGC ACA GTC ATT GAA AG), IFN-γ *reverse* (AAT CTG GCT CTG CAG GAT TT), GAPDH *forward* (GAG GCC GGT GCT GAG TAT GT) and GAPDH *reverse* (GGT GGC AGT GAT GGC ATG GA). Tenfold serial dilution standards ranging from 10^8^ to 10^2^ copies/μL were used for quantification and the specificity of each reaction was controlled by melting curve analysis. GAPDH was used as housekeeping gene to normalize the results.

### 2.5 Plaque assay

Plaque assay was performed as described (60). Briefly, a frozen section of cerebral tissue was weighted, diluted in DMEM to a concentration of 10%, and homogenized using an Omni Tissue Homogenizer (Süd-Laborbedarf GmbH). Then, serial dilutions of homogenates were added to 6-well culture plates (Merck KGaA, Darmstadt, Germany) containing a confluent layer of L cells for 1 hour at room temperature. Subsequently, cells were covered with methyl cellulose (Merck) and incubated for 72 hours at 37°C. Finally, cells were fixed with 10% buffered formalin and stained with crystal violet (Merck) to allow counting of plaque forming units (PFU).

### 2.6 Flow cytometry

Spleens and brains were dissected from TMEV- or mock-infected C57BL/6 *Glb1^+/+^* and homozygous *Glb1^-/-^* mice and stored in IMDM complete (IMDM, 10% FCS, 2 mM L-glu, 100 U/ml penicillin, 100 µg/ml streptomycin) until further processing. For processing, brain isolates (left front quarter of cerebrum) were transferred to a petri dish filled with IMDM complete and dissociated using the back of a 25 ml syringe (Roth, #0058.1). For further homogenization, cell dissociates were re-suspended several times. All isolated cells were filtrated using a cell strainer (40 µm) to obtain a uniform single-cell suspension. The filtered cells were centrifuged (300 x g, 5 min, 4°C), the supernatant was discarded and RBCs in the pellet were lysed in lysis buffer (10% 100 mM Tris pH 7.5, 90% 160 mM NH_4_Cl) for 5 min at room temperature (RT). Cells were centrifuged twice with one PBS-washing step in between and the resulting pelleted cells were resuspended in IMDM complete until flow cytometrical staining. For flow cytometrical staining, all cells were blocked with anti-mouse CD16/32 (93, eBioscience) for 10 min at 4°C. Afterwards, splenocytes and brain cells were stained for composition and activation state. To analyse splenocyte composition, cells were either stained with PE-conjugated anti-mouse CD4 (GK1.5, BD Biosciences, Franklin Lakes, NJ), APC-conjugated anti-mouse CD8a (53-6.7, BD Biosciences) and FITC-conjugated anti-mouse CD19 (1D3, eBioscience, Frankfurt am Main, Germany) or APC-conjugated anti-mouse CD11c (N418, eBioscience), PE-conjugated anti-mouse CD11b (M1/70, BD Biosciences) and FITC-conjugated anti-mouse CD19 (1D3, eBioscience, Frankfurt am Main, Germany) for 20 min at 4°C. To analyse brain cell composition, cells were stained with PerCP-Cy5.5-conjugated anti-mouse CD45 (30-F11, eBioscience, Frankfurt am Main, Germany), APC-conjugated anti-mouse CD11c (N418, eBioscience) and PE-conjugated anti-mouse CD11b (M1/70, BD Biosciences) for 20 min at 4°C. To analyse the activation of CD4 and CD8a positive T cells in spleen and brain, cells were either stained with FITC-conjugated anti-mouse CD4 (RM4-5, eBioscience), PE-Cy7-conjugated anti-mouse CD62L (MEL-14, eBioscience) and APC-conjugated anti-mouse CD69 (H1.2F3, eBioscience) or FITC-conjugated anti-mouse CD8a (53-6.7, eBioscience), PE-Cy7-conjugated anti-mouse CD62L (MEL-14, eBioscience) and APC-conjugated anti-mouse CD69 (H1.2F3, eBioscience), respectively, for 20 min at 4°C. All spleen and brain samples were analysed using an Attune NxT Flow Cytometer (Thermo Fisher Scientific, Waltham, MA). The gating strategy is illustrated in the **Supplementary Figure 1**. Data analysis was performed using FlowJo (Version 10, FlowJo LLC., Ashland, OR). Due to variability of sample size, cell numbers were normalized to 10^7^ analyzed cells.

### 2.7 Statistics, graphs and figures

Statistical analysis was performed using SAS/STAT software, Version 7.1 of the SAS^®^ System for Windows (SAS Institute Inc.). Graph plotting was performed using GraphPad Prism 9. Significance values for clinical, histological and immunohistochemical results, except for TMEV, were compared via Wilcoxon Tests with Dwass-Steel-Critchlow-Fligner method as *post-hoc* tests. Similarly, RT-qPCR and plaque assay data were analysed using Wilcoxon Tests. Both the immunohistochemical results for TMEV as well as the flow cytometry-based assays were compared via Mann-Whitney-U-Test including Bonferroni-correction for the latter. Figures for clinical, histological and immunohistochemical data were generated using GIMP 2.10.32.

## 3 Results

### 3.1 Increased virus replication and delayed inflammatory response in *Glb1^-/-^* mice after TMEV infection

LSDs can affect immune responses, even before the onset of clinical disease. Thus, antiviral immune responses might be restricted in *Glb1* ^-/-^ mice making them susceptible to TMEV-IDD. To investigate changes in the susceptibility of *Glb1* ^-/-^ mice to TMEV-IDD, we infected *Glb1^-/-^* and *Glb1^+/+^* control mice on a C57BL/6 background with the BeAn strain of TMEV.

**Figure 1:**
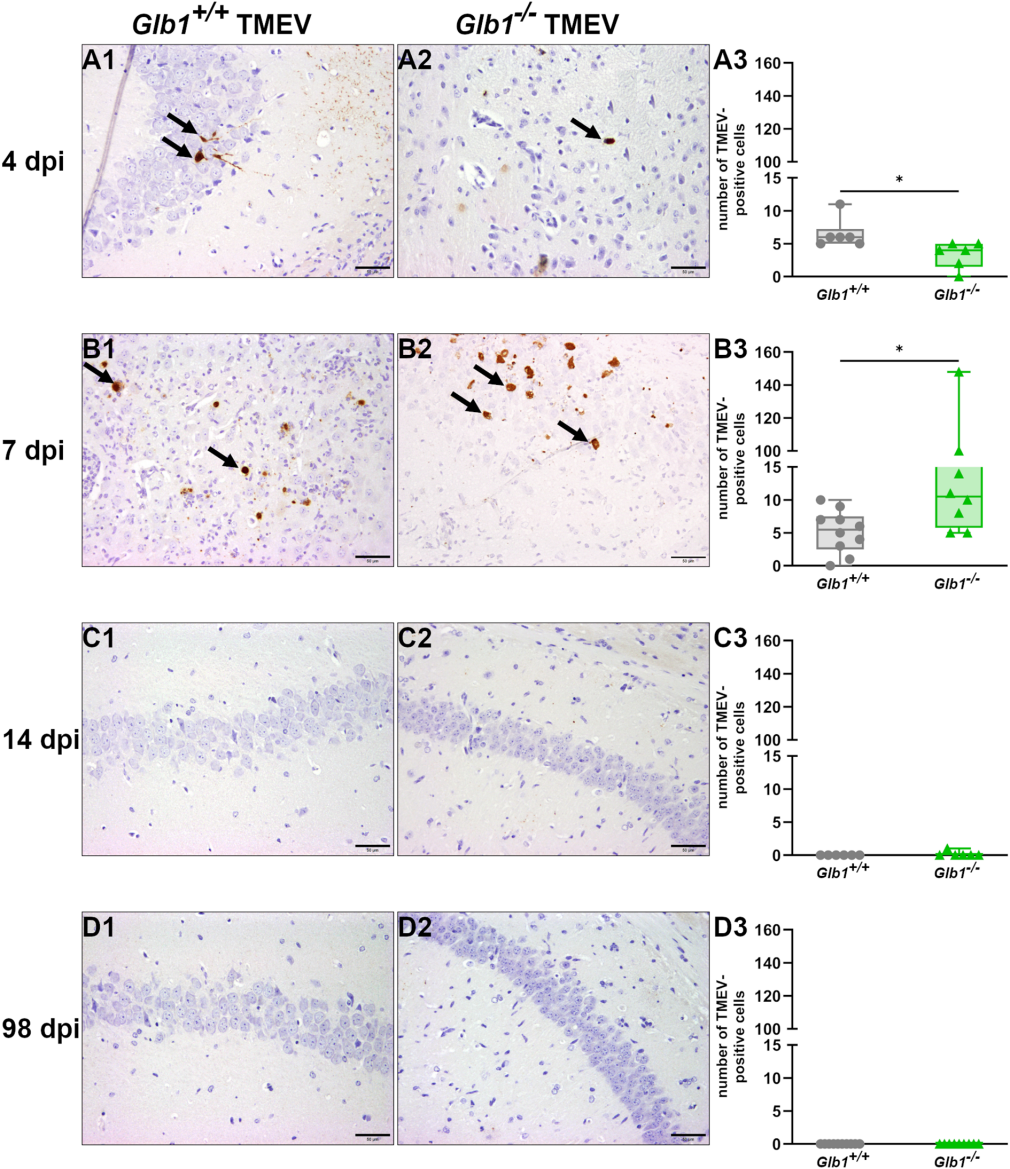
Total number of Theiler’s murine encephalomyelitis virus (TMEV)-antigen positive cells per brain section of TMEV-infected *Glb1^-/-^* and C57BL/6 wildtype (*Glb1^+/+^*) mice at 4, 7, 14, and 98 days post infection (dpi). **(A1-A3)** At 4 dpi, *Glb1^+/+^* mice (grey dots) displayed a significant increase in the number of TMEV-positive cells in the brain compared to *Glb1^-/-^* mice (green triangles, p=0.013). **(B1-B3)** At 7 dpi, *Glb1^-/-^* mice (green triangles) showed a significantly higher number of TMEV-positive cells in the brain compared to *Glb1^+/+^* mice (grey dots, p=0.016). **(C1-C3, D1-D3)** At 14 and 98 dpi, neither *Glb1^-/-^* mice (green triangles) nor *Glb1^+/+^* mice (grey dots) showed TMEV-positive cells in the brain. Box and whisker plots (min-max) with medians and all data points. ABC-DAB immunohistochemistry, TMEV; Positive cells (→). Bars (A1-D2) = 50 µm. Images taken from hippocampus. 4 dpi: *Glb1^-/-^* TMEV: n=6, *Glb1^-/-^* Placebo: n=6, *Glb1^+/+^* TMEV: n=6, *Glb1^+/+^* Placebo: n=6; 7 dpi: *Glb1^-/-^* TMEV: n=8, *Glb1^-/-^* Placebo: n=8, *Glb1^+/+^* TMEV: n=10, *Glb1^+/+^* Placebo: n=10; 14 dpi: *Glb1^-/-^* TMEV: n=6, *Glb1^-/-^* Placebo: n=6, *Glb1^+/+^* TMEV: n=6, *Glb1^+/+^* Placebo: n=6; 98 dpi: *Glb1^-/-^* TMEV: n=9, *Glb1^-/-^* Placebo: n=5, *Glb1^+/+^* TMEV: n=10, *Glb1^+/+^* Placebo: n=10.

Immunohistochemistry was used to localize and quantify TMEV antigen in the brain. Moreover, virus load in the brain was quantified via RT-qPCR and plaque assay. At 4 dpi, a slightly higher number of TMEV-positive cells was found in *Glb1^+/+^* compared to *Glb1* ^-/-^ mice via immunohistochemistry, but generally only few cells contained viral antigen (**Figure 1A1-A3**). RT-qPCR and plaque assay showed a similar trend towards a higher virus load in *Glb1^+/+^* mice at 4 dpi but a statistically significant difference was lacking (**Supplementary Figure 2**). At 7 dpi, *Glb1* ^-/-^ mice showed more TMEV-positive cells than *Glb1^+/+^* mice via immunohistochemistry, which is consistent with a retarded viral clearance (**Figure 1B1-B3**). RT-qPCR and plaque assay also demonstrated a trend for a higher viral load in Glb1^-/-^ mice at 7 dpi but no statistically significant difference was found (**Supplementary Figure 2**). At 14 and 98 dpi, neither *Glb1* ^-/-^ nor *Glb1^+/+^* mice displayed TMEV-positive cells in the brain (**Figure 1C1-D3**). However, RT-qPCR still detected TMEV RNA in two *Glb1* ^-/-^ mice at 14 dpi (**Supplementary Figure 2**).

**Figure 2:**
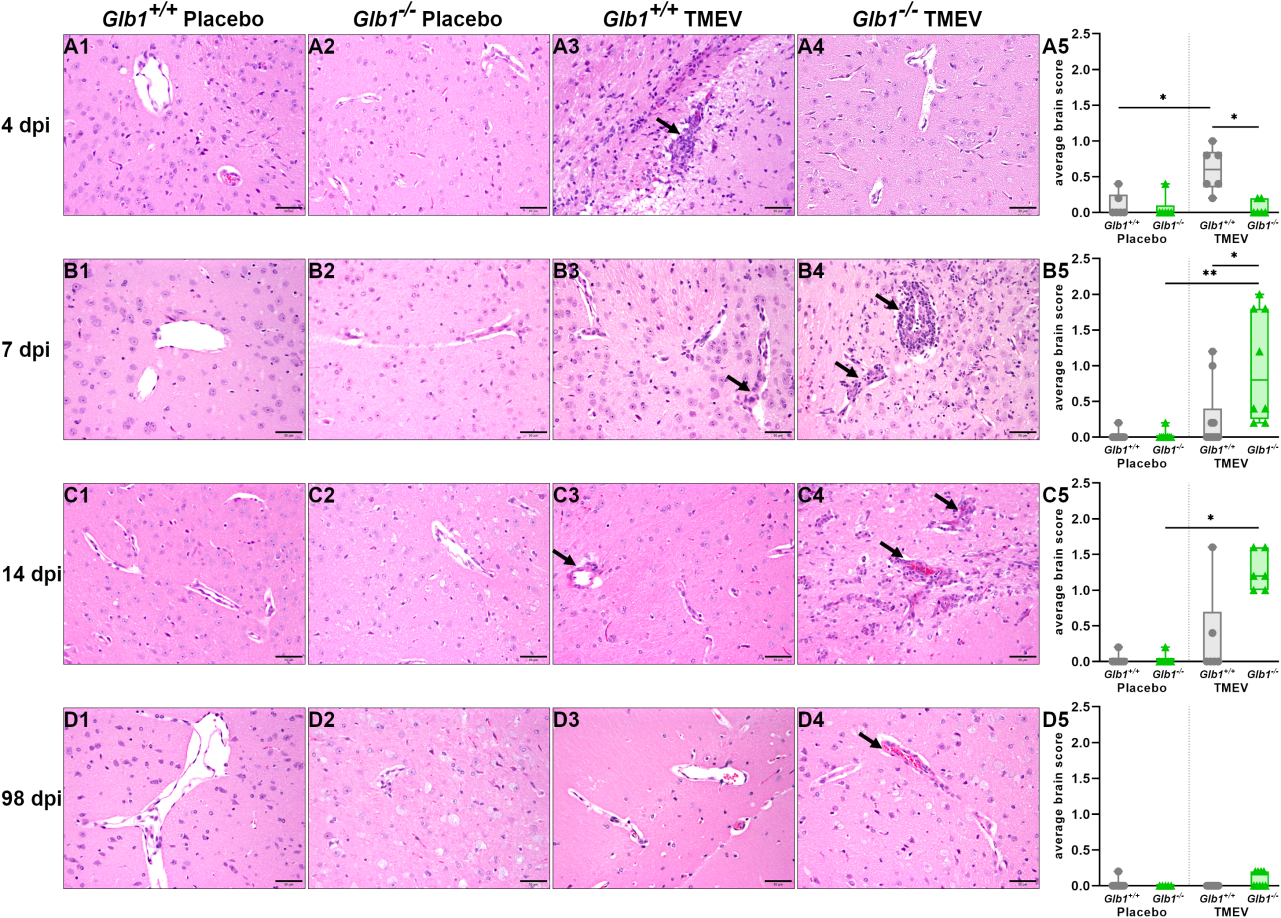
Average semiquantitative score for perivascular cuffing in the whole brain sections of Theiler’s murine encephalomyelitis virus (TMEV)- and mock-infected *Glb1^-/-^* and C57BL/6 wildtype (*Glb1^+/+^*) mice at 4, 7, 14, and 98 days post infection (dpi). **(A1-A5)** At 4 dpi, TMEV-infected *Glb1^+/+^* mice (right, grey dots) displayed significantly increased perivascular cuffing compared to mock-infected *Glb1^+/+^* mice (left, grey dots, p=0.047) and TMEV-infected *Glb1^-/-^* mice (right, green triangles, p=0.026). **(B1-B5)** At 7 dpi, TMEV-infected *Glb1^-/-^* mice (right, green triangles) showed a significant increase in perivascular cuffing compared to both mock-infected *Glb1^-/-^* mice (left, green triangles, p=0.0036) and TMEV-infected *Glb1^+/+^* mice (right, grey dots, p=0.036). **(C1-C5)** At 14 dpi, TMEV-infected *Glb1^-/-^* mice (right, green triangles) displayed a significant increase in perivascular cuffing compared to mock-infected *Glb1^-/-^* mice (left, green triangles, p=0.014). **(D1-D5)** At 98 dpi, overall perivascular leukocyte infiltration was very low without significant differences between the study groups. Box and whisker plots (min-max) with medians and all data points. Hematoxylin & Eosin staining; Positive cells (→). Bars (A1-D4) = 50 µm. Images taken from diencephalon/mesencephalon. 4 dpi: *Glb1^-/-^* TMEV: n=6, *Glb1^-/-^* Placebo: n=6, *Glb1^+/+^* TMEV: n=6, *Glb1^+/+^* Placebo: n=6; 7 dpi: *Glb1^-/-^* TMEV: n=8, *Glb1^-/-^* Placebo: n=8, *Glb1^+/+^* TMEV: n=10, *Glb1^+/+^* Placebo: n=10; 14 dpi: *Glb1^-/-^* TMEV: n=6, *Glb1^-/-^* Placebo: n=6, *Glb1^+/+^* TMEV: n=6, *Glb1^+/+^* Placebo: n=6; 98 dpi: *Glb1^-/-^* TMEV: n=9, *Glb1^-/-^* Placebo: n=5, *Glb1^+/+^* TMEV: n=10, *Glb1^+/+^* Placebo: n=10.

Next, to investigate the impact of G_M1_ gangliosidosis on inflammatory processes in the brain, perivascular cuffing was quantified using a semiquantitative scoring system. As expected, at 4 dpi, stronger perivascular cuffing was found in TMEV- compared to mock-infected *Glb1^+/+^* mice. Interestingly, TMEV-infected *Glb1^+/+^* mice also exhibited a stronger perivascular cuffing than TMEV-infected *Glb1* ^-/-^ mice (**Figure 2A1-A5**). In contrast, at 7 dpi, TMEV-infected *Glb1* ^-/-^ mice displayed a stronger perivascular cuffing than mock-infected *Glb1* ^-/-^ mice and TMEV-infected *Glb1^+/+^* mice (**Figure 2 B1-B5**). At 14 dpi, a stronger perivascular cuffing was present in TMEV-compared to mock-infected *Glb1* ^-/-^ mice, whereas no significant difference was found between TMEV-infected *Glb1^+/+^* and *Glb1* ^-/-^ mice (**Figure 2C1-C5**). Cortex, hippocampus and diencephalon/mesencephalon (close to the injection site) were most strongly affected by perivascular cuffing. Finally, perivascular cuffing was low or even absent in mice at 98 dpi without statistically significant differences between the groups (**Figure 2D1-D5**). This suggests that *Glb1^-/-^* mice mount a delayed inflammatory response to TMEV-infection, which is still present at 14 dpi in the brain. Corresponding to the lack of increased clinical disease in TMEV-infected animals, no inflammatory or demyelinating lesions were present in the spinal cord of TMEV- and mock-infected *Glb1* ^-/-^ and *Glb1^+/+^* mice at 98 dpi.

### 3.2 Delayed T cell infiltration into the brain in *Glb1^-/-^* mice after TMEV infection

**Figure 3:**
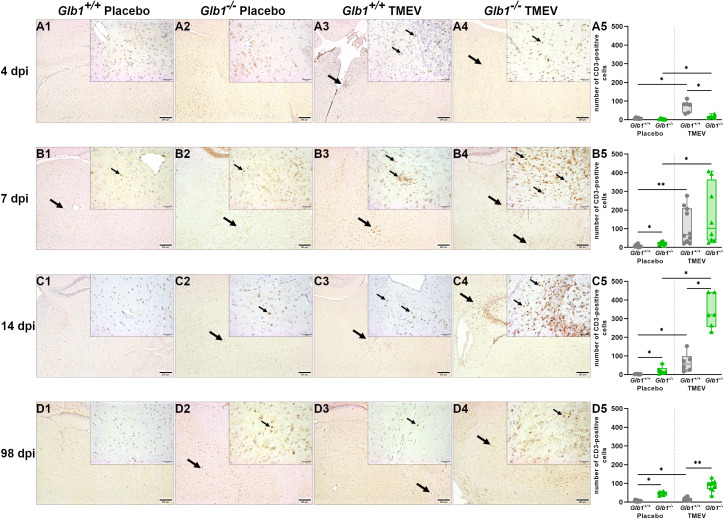
Total number of CD3-positive T cells per brain section of Theiler’s murine encephalomyelitis virus (TMEV)- and mock-infected *Glb1^-/-^* and C57BL/6 wildtype (*Glb1^+/+^*) mice at 4, 7, 14, and 98 days post infection (dpi). **(A1-A5)** At 4 dpi, TMEV-infected *Glb1^+/+^* mice (right, grey dots) displayed a significant increase in CD3^+^ T cells compared to mock-infected *Glb1^+/+^* mice (left, grey dots, p=0.02) and TMEV-infected *Glb1^-/-^* mice (right, green triangles, p=0.025). TMEV-infected *Glb1^-/-^* mice (right, green triangles) showed an increased number of CD3^+^ T cells compared to mock-infected (left, green triangles) *Glb1^-/-^* mice (p=0.049). **(B1-B5)** At 7 dpi, TMEV-infected *Glb1^+/+^* mice (right, grey dots) displayed a significant increase in CD3^+^ T cells compared to mock-infected *Glb1^+/+^* mice (left, grey dots, p=0.003). Similarly, TMEV-infected *Glb1^-/-^* mice (right, green triangles) showed a significantly increased number of CD3^+^ T cells compared to mock-infected *Glb1^-/-^* mice (left, green triangles, p=0.015). Mock-infected *Glb1^-/-^* mice (left, green triangles) also displayed a significantly increased number of CD3^+^ T cells in the brain compared to mock-infected *Glb1^+/+^* mice (left, grey dots, p=0.017). **(C1-C5)** At 14 dpi, TMEV-infected *Glb1^-/-^* mice (right, green triangles) displayed a significantly increased number of CD3^+^ T cells compared to mock-infected *Glb1^-/-^* mice (left, green triangles, p=0.02) and TMEV-infected *Glb1^+/+^* mice (right, grey dots, p=0.02). TMEV-infected *Glb1^+/+^* mice (right, grey dots) also showed significantly increased CD3^+^ T cells compared to mock-infected *Glb1^+/+^* mice (left, grey dots, p=0.019). In addition, mock-infected *Glb1^-/-^* mice (left, green triangles) displayed a significantly increased number of CD3^+^ T cells in the brain compared to mock-infected *Glb1^+/+^* mice (left, grey dots, p=0.019). **(D1-D5)** At 98 dpi, TMEV-infected *Glb1^-/-^* mice (right, green triangles) displayed significantly increased CD3^+^ T cells compared to TMEV-infected *Glb1^+/+^* mice (right, grey dots, p=0.0014) and mock-infected *Glb1^-/-^* mice (left, green triangles) showed significantly increased CD3^+^ T cells compared to mock-infected *Glb1^+/+^* mice (left, grey dots, p=0.012). TMEV-infected *Glb1^+/+^* mice (right, grey dots) also showed an increased number of CD3^+^ T cells compared to mock-infected *Glb1^+/+^* mice (left, grey dots, p=0.015). Box and whisker plots (min-max) with medians and all data points. ABC-DAB immunohistochemistry, CD3; Positive cells (→). Bars (A1-D4) = 200 µm, bars in inserts (A1-D4) = 50 µm. Overview images taken from hippocampus and diencephalon/mesencephalon, insert images taken from diencephalon/mesencephalon (C4 insert image taken from hippocampus). 4 dpi: *Glb1^-/-^* TMEV: n=6, *Glb1^-/-^* Placebo: n=6, *Glb1^+/+^* TMEV: n=6, *Glb1^+/+^* Placebo: n=6; 7 dpi: *Glb1^-/-^* TMEV: n=8, *Glb1^-/-^* Placebo: n=8, *Glb1^+/+^* TMEV: n=10, *Glb1^+/+^* Placebo: n=10; 14 dpi: *Glb1^-/-^* TMEV: n=6, *Glb1^-/-^* Placebo: n=6, *Glb1^+/+^* TMEV: n=6, *Glb1^+/+^* Placebo: n=6; 98 dpi: *Glb1^-/-^* TMEV: n=9, *Glb1^-/-^* Placebo: n=5, *Glb1^+/+^* TMEV: n=10, *Glb1^+/+^* Placebo: n=10.

**Figure 4:**
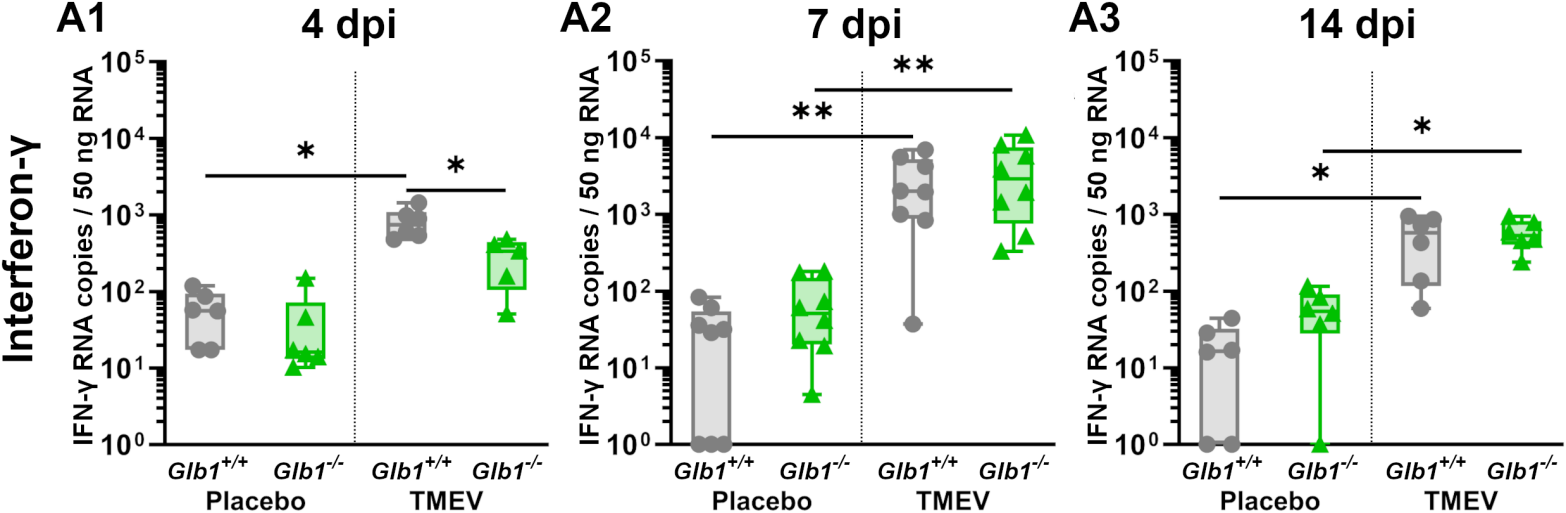
Interferon-γ (IFN-γ) levels in the brain of of Theiler’s murine encephalomyelitis virus (TMEV)- and mock-infected *Glb1^-/-^* and C57BL/6 wildtype (*Glb1^+/+^*) mice at 4, 7 and 14 days post infection (dpi). **(A1)** 4 dpi: TMEV-infected *Glb1^+/+^* mice display higher IFN-γ levels than both TMEV-infected *Glb1^-/-^* mice (p=0.03) and mock-infeckted *Glb1^+/+^* mice (p=0.02). **(A2)** 7 dpi: Both TMEV-infected *Glb1^+/+^* mice and TMEV-infected *Glb1^-/-^* mice displayed higher IFN-γ levels than their respective mock-infected control group (*Glb1^+/+^*: p=0.009; *Glb1^-/-^*: p=0.004). **(A3)** 14 dpi: Both TMEV-infected *Glb1^+/+^* mice and TMEV-infected *Glb1^-/-^* mice displayed lower IFN-γ levels than at 7 dpi, but still higher than their respective mock-infected control group (*Glb1^+/+^*: p=0.02; *Glb1^-/-^*: p=0.02). Box and whisker plots (min-max) with medians and all data points. *: p<0.05; **: p<0.01. 4 dpi: *Glb1^-/-^* TMEV: n=5, *Glb1^-/-^* Placebo: n=6, *Glb1^+/+^* TMEV: n=6, *Glb1^+/+^* Placebo: n=6; 7 dpi: *Glb1^-/-^* TMEV: n=8, *Glb1^-/-^* Placebo: n=8, *Glb1^+/+^* TMEV: n=8, *Glb1^+/+^* Placebo: n=8; 14 dpi: *Glb1^-/-^* TMEV: n=6, *Glb1^-/-^* Placebo: n=6, *Glb1^+/+^* TMEV: n=6, *Glb1^+/+^* Placebo: n=6.

T cell infiltration into the CNS was further assessed by quantifying CD3^+^ cells. At 4 dpi, TMEV-infected *Glb1^+/+^* mice displayed a higher number of CD3^+^ T cells than TMEV-infected *Glb1^-/-^* mice. (**Figure 3A1-A5**). At 7 dpi, TMEV infection resulted in an increase in T cell numbers in both *Glb1^+/+^* and *Glb1* ^-/-^ mice, which remained at a higher level in *Glb1* ^-/-^ compared to *Glb1^+/+^* mice at 14 and 98 dpi (**Figure 3B1-D5**). At 98 dpi, only *Glb1^-/-^* but not *Glb1^+/+^* mice showed an increased number of CD3^+^ T cells after TMEV infection (**Figure 3D1-D5**). From 7 dpi until 98 dpi, mock-infected *Glb1^-/-^* mice displayed increased T cell numbers in the brain compared to mock-infected *Glb1^+/+^* mice (**Figure 3B1-D5**). Overall, these data indicate a delayed antiviral T cell response in *Glb1* ^-/-^ compared to *Glb1^+/+^* mice after TMEV infection but a slightly enhanced T cell infiltration into the CNS due to G_M1_ lysosomal storage in older animals. Additionally, to evaluate lymphocyte activity (especially CD8^+^ T cells, but also NK cells), IFN-γ levels were measured at 4, 7 and 14 dpi. Similar to the immunohistochemical results (**Figure 3**), TMEV-infected *Glb1^+/+^* mice displayed higher levels of IFN-γ than TMEV-infected *Glb1^-/-^* mice at 4 dpi (**Figure 4**). At 7 and 14 dpi, TMEV-infected *Glb1^+/+^* mice and TMEV-infected *Glb1^-/-^* mice displayed equally higher levels of IFN-γ than their respective mock-infected control groups (**Figure 4**).

**Figure 5:**
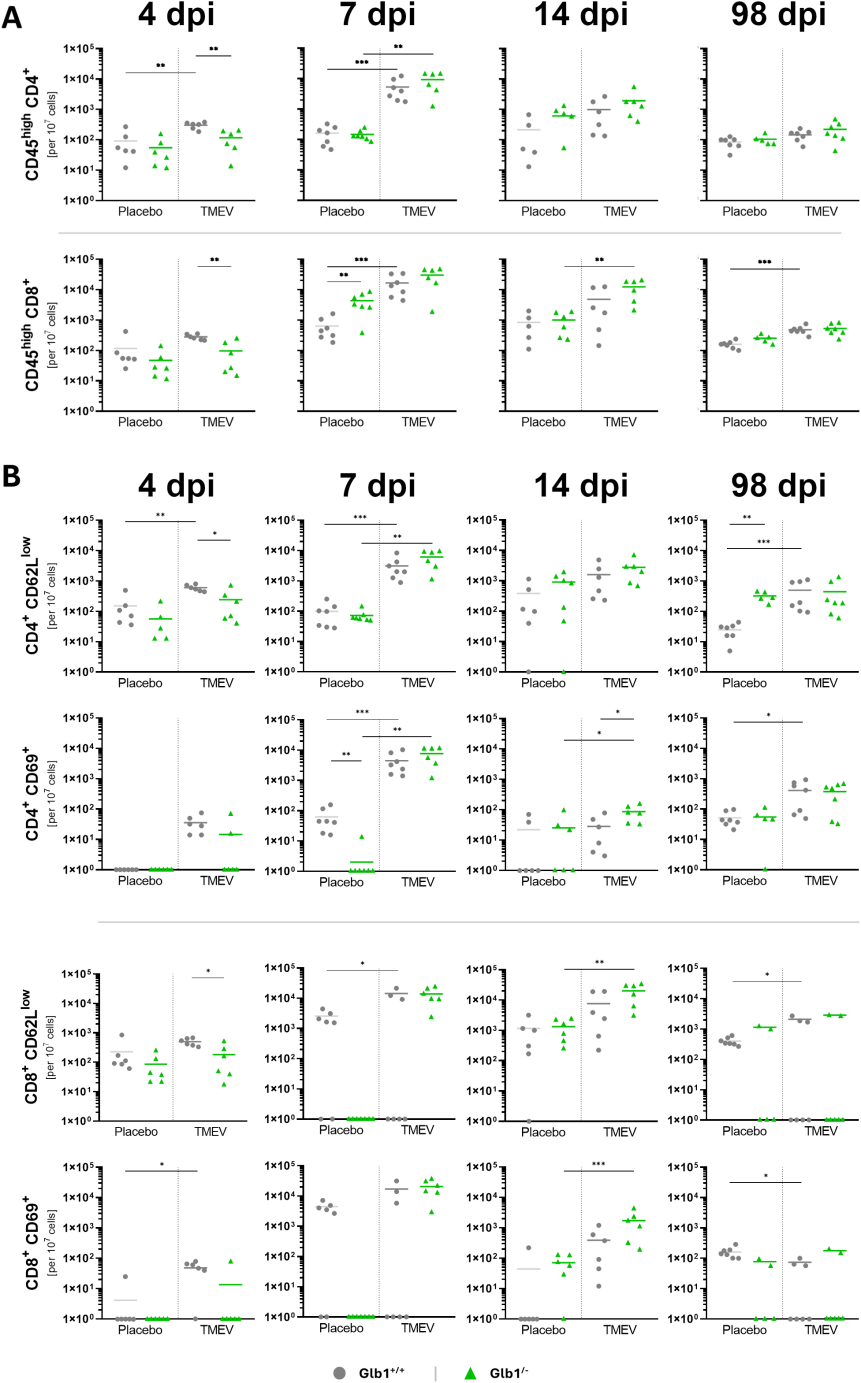
Flow cytometry of cells isolated from brains of Theiler’s murine encephalomyelitis virus (TMEV)- and mock-infected *Glb1^-/-^* and C57BL/6 wildtype (*Glb1^+/+^*) mice at 4, 7, 14, and 98 days post infection (dpi). Total cell numbers of activated CD4^+^ and CD8^+^ T cells **(A)** based on CD62L^low^ and CD69 **(B)**, respectively (rows, from top to bottom) at 4, 7, 14, and 98 dpi (columns, from left to right). In general, *Glb1^-/-^* mice displayed a delayed T cell response during TMEV infection compared to *Glb1^+/+^* mice. Means with all data points. *p < 0.05, **p < 0.01, ***p < 0.001 with Bonferroni-correction. 4 dpi: *Glb1^-/-^* TMEV: n=6, *Glb1^-/-^* Placebo: n=6, *Glb1^+/+^* TMEV: n=6, *Glb1^+/+^* Placebo: n=6; 7 dpi: *Glb1^-/-^* TMEV: n=6, *Glb1^-/-^* Placebo: n=7, *Glb1^+/+^* TMEV: n=7, *Glb1^+/+^* Placebo: n=7; 14 dpi: *Glb1^-/-^* TMEV: n=6, *Glb1^-/-^* Placebo: n=6, *Glb1^+/+^* TMEV: n=6, *Glb1^+/+^* Placebo: n=6; 98 dpi: *Glb1^-/-^* TMEV: n=7, *Glb1^-/-^* Placebo: n=5, *Glb1^+/+^* TMEV: n=7, *Glb1^+/+^* Placebo: n=7.

Subsequently, flow cytometry was used to assess both the composition and activation of CD4^+^ and CD8^+^ T cells in the brain during TMEV infection. In general, the most prominent effects were observed at 4 and 7 dpi. At 4 dpi, TMEV-infected *Glb1^+/+^* mice already exhibited an increased number of CD4^+^ and CD8^+^ T cells (**Figure 5A**). Only starting at 7 dpi, these changes were observed for cells from *Glb1^-/-^* mice, while both *Glb1^+/+^* and *Glb1^-/-^* mice showed robust T cell activation as indicated by the down-regulated CD62L expression and increased CD69 expression by both CD4^+^ and CD8^+^ T cells (**Figure 5B**). From 14 dpi on, the number and activation of brain-infiltrated T cells started to decline (**Figure 5**). This decline was more prominent in *Glb1^+/+^* mice, which again indicates a protracted response in *Glb1^-/-^* mice as seen in immunohistochemistry. At 98 dpi, immune cell composition in the brain and their activation had largely returned to basal levels, comparable to mock-infected *Glb1^+/+^* and *Glb1^-/-^* mice (**Figure 5**). In the spleen, there was a tendency (sometimes significant) for a higher frequency of CD4^+^ in the *Glb1^+/+^* mice, which could reflect a larger T cell repertoire in comparison to the knockout mice (**Supplementary Figures 3, 4**).

### 3.3 Enhanced microglia/macrophage activation in *Glb1* ^-/-^ mice after TMEV infection

**Figure 6:**
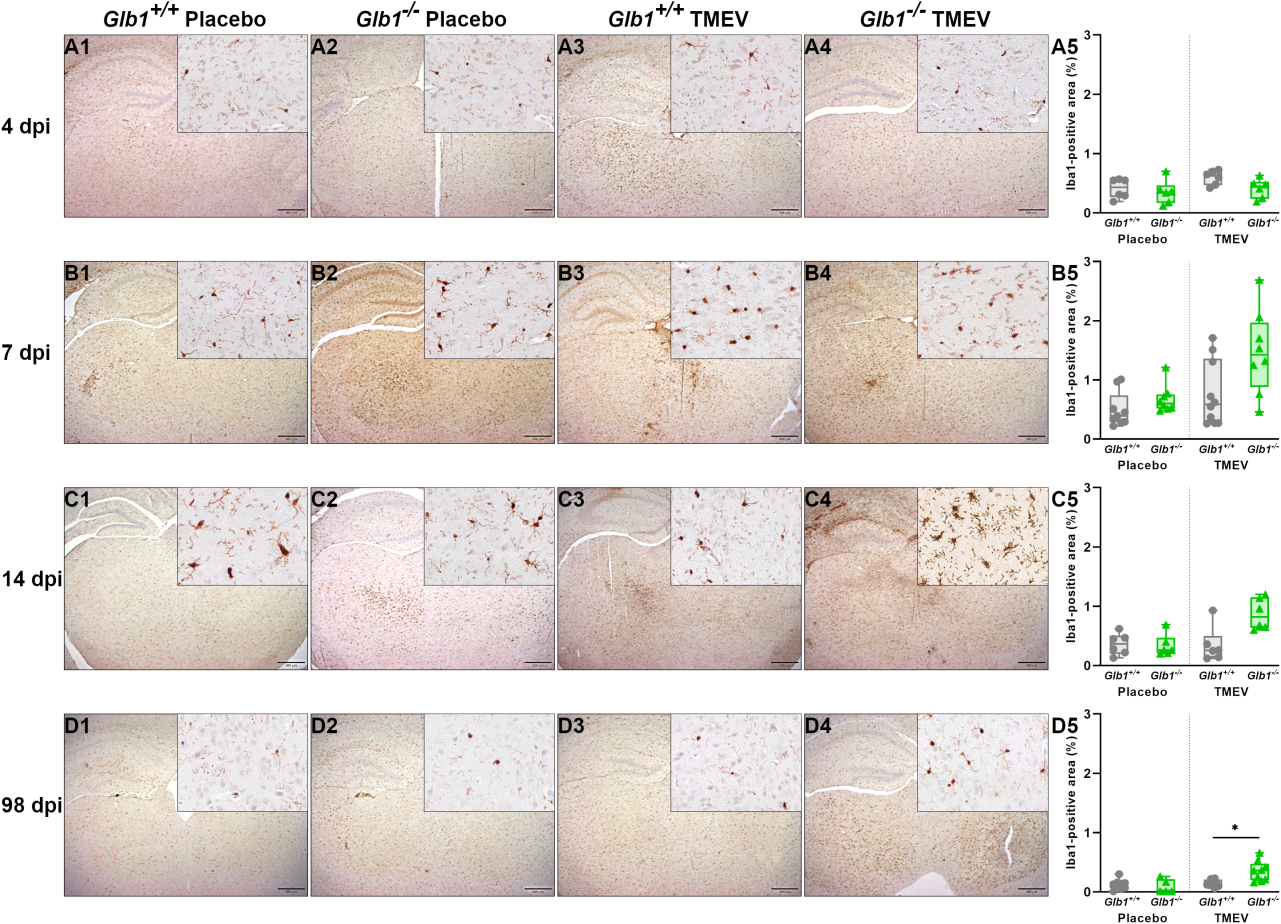
Relative area of Iba1-positive microglia/macrophages pere brain section of Theiler’s murine encephalomyelitis virus (TMEV)- and mock-infected *Glb1^-/-^* and C57BL/6 wildtype (*Glb1^+/+^*) mice at 4, 7, 14, and 98 days post infection (dpi). **(A1-A5, B1-B5, C1-C5)** At 4, 7 and 14 dpi, there were no significant differences between the study groups regarding the Iba1^+^ area. **(D1-D5)** At 98 dpi, TMEV-infected *Glb1^-/-^* mice (right, green triangles) showed a significant increase in Iba1^+^ area compared to TMEV-infected *Glb1^+/+^* mice (right, grey dots, p=0.015). Box and whisker plots (min-max) with medians and all data points. ABC-DAB immunohistochemistry, Iba1; Bars (A1-D4) = 500 µm. Overview images taken from hippocampus and diencephalon/mesencephalon, insert images taken from diencephalon/mesencephalon. 4 dpi: *Glb1^-/-^* TMEV: n=6, *Glb1^-/-^* Placebo: n=6, *Glb1^+/+^* TMEV: n=6, *Glb1^+/+^* Placebo: n=6; 7 dpi: *Glb1^-/-^* TMEV: n=8, *Glb1^-/-^* Placebo: n=8, *Glb1^+/+^* TMEV: n=10, *Glb1^+/+^* Placebo: n=10; 14 dpi: *Glb1^-/-^* TMEV: n=6, *Glb1^-/-^* Placebo: n=6, *Glb1^+/+^* TMEV: n=6, *Glb1^+/+^* Placebo: n=6; 98 dpi: *Glb1^-/-^* TMEV: n=9, *Glb1^-/-^* Placebo: n=5, *Glb1^+/+^* TMEV: n=10, *Glb1^+/+^* Placebo: n=10.

To quantify the expansion of microglia/macrophages in the brain, we stained for Iba1 and determined the immunopositive area using morphometry. Furthermore, the perivascular infiltration of macrophages in the early phase of the disease (4 and 7 dpi) was semiquantitatively scored on sections stained for Iba1. At 4, 7 and 14 dpi, there were no statistically significant differences in the relative Iba1-positive area between the study groups (**Figure 6A1-C5**). In contrast, at 98 dpi, TMEV-infected *Glb1* ^-/-^ mice displayed an increase in the Iba1-positive area compared to TMEV-infected *Glb1^+/+^* mice indicating a long-lasting effect of TMEV infection on the number and/or size of microglia/macrophages in the brain (maybe due to a transition from a ramified to a hyperramified or amoeboid state of microglial cells), which seems to be enhanced by G_M1_ lysosomal storage (**Figure 6C1-D5**). Moreover, the evaluation of perivascular Iba1-positive cells at 4 and 7 dpi showed a delayed infiltration of macrophages into the CNS of TMEV-infected *Glb1^-/-^* mice compared to TMEV-infected *Glb1^+/+^* mice similar to the delayed infiltration of T cells described above (**Supplementary Figure 5**).

Flow cytometry was also used to assess the numbers of antigen-presenting cells in the brain during TMEV infection. Only minor changes were found for the absolute numbers of CD45^int^CD11b^+^ cells (predominantly microglia). Similar to T cells, increased numbers of CD45^high^CD11b^+^ (most likely macrophage but also natural killer (NK) cell) and CD45^high^CD11c^+^ (most likely dendritic cell) populations were found in TMEV-infected *Glb1^+/+^* mice already at 4 dpi and in *Glb1^-/-^* mice not until 7 dpi (**Supplementary Figure 6**). At later time points, the total number of CD45^high^CD11b^+^ and CD45^high^CD11c^+^ cells in the brain returned to basal levels comparable to mock-infected *Glb1^+/+^* and *Glb1^-/-^* mice (**Supplementary Figure 6**).

**Figure 7:**
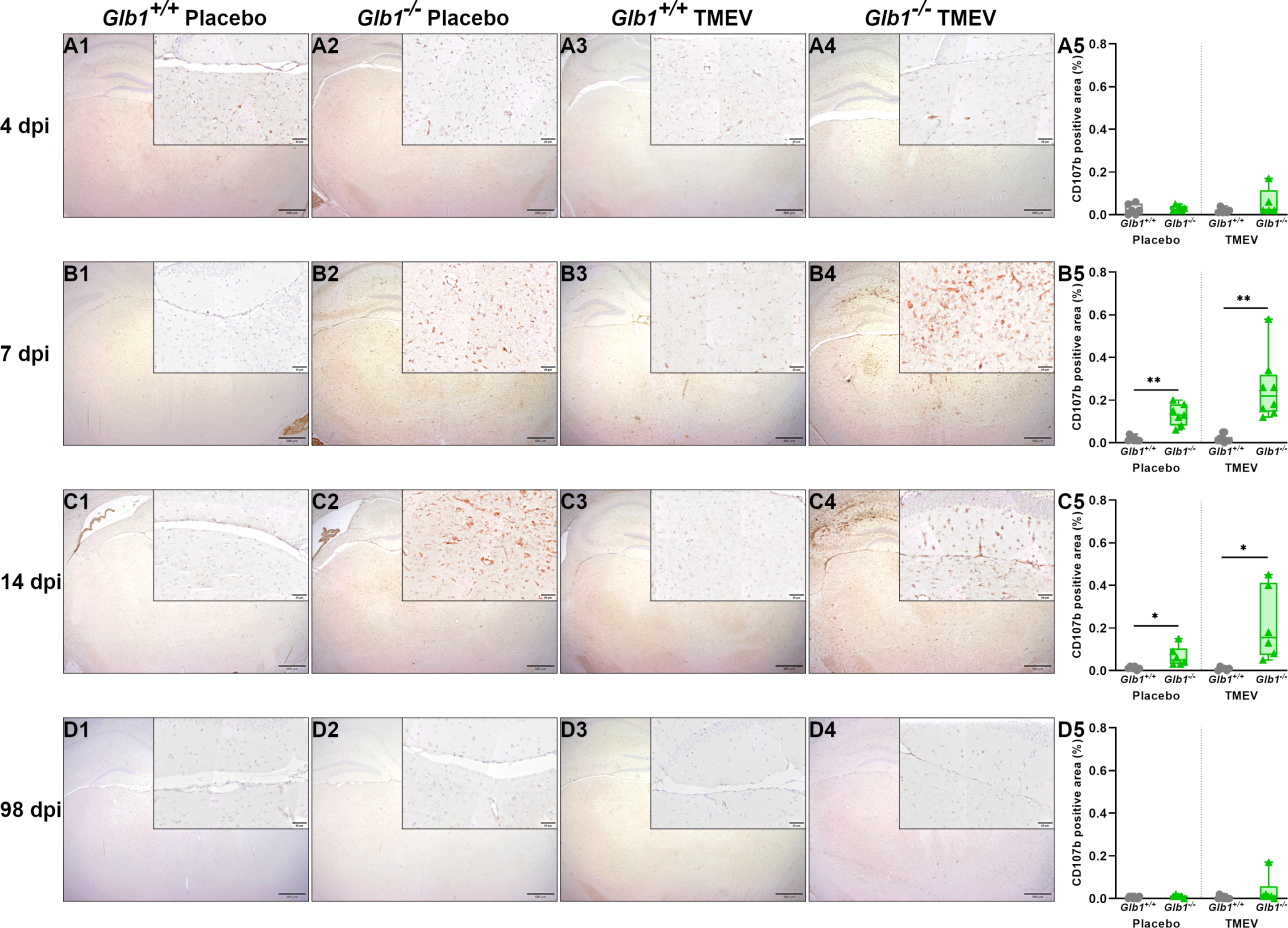
Relative area of CD107b-positive microglia/macrophages per brain section of Theiler’s murine encephalomyelitis virus (TMEV)- and mock-infected *Glb1^-/-^* and C57BL/6 wildtype (*Glb1^+/+^*) mice at 4, 7, 14, and 98 days post infection (dpi). **(A1-A5)** At 4 dpi, there were no significant differences between the study groups regarding the CD107b^+^ area. **(B1-B5)** At 7 dpi, TMEV-infected *Glb1^-/-^* mice (right, green triangles) displayed a significant increase in CD107b^+^ area compared to TMEV-infected *Glb1^+/+^* mice (right, grey dots, p=0.002). Additionally, mock-infected *Glb1^-/-^* mice (left, green triangles) displayed a significant increase in CD107b^+^ area compared to mock-infected *Glb1^+/+^* mice (left, grey dots, p=0.004). **(C1-C5)** At 14 dpi, similarly, TMEV-infected *Glb1^-/-^* mice (right, green triangles) displayed a significant increase in CD107b^+^ area compared to TMEV-infected *Glb1^+/+^* mice (right, grey dots, p=0.02) and mock-infected *Glb1^-/-^* mice (left, green triangles) displayed a significant increase in CD107b^+^ area compared to mock-infected *Glb1^+/+^* mice (left, grey dots, p=0.02). **(D1-D5)** At 98 dpi, there were no significant differences between the study groups regarding the CD107b^+^ area. Box and whisker plots (min-max) with medians and all data points. ABC-DAB immunohistochemistry, CD107b; Bars **(A1-D4)** = 500 µm. Overview images taken from hippocampus and diencephalon/mesencephalon, insert images taken from diencephalon/mesencephalon. 4 dpi: *Glb1^-/-^* TMEV: n=6, *Glb1^-/-^* Placebo: n=6, *Glb1^+/+^* TMEV: n=6, *Glb1^+/+^* Placebo: n=6; 7 dpi: *Glb1^-/-^* TMEV: n=8, *Glb1^-/-^* Placebo: n=8, *Glb1^+/+^* TMEV: n=10, *Glb1^+/+^* Placebo: n=10; 14 dpi: *Glb1^-/-^* TMEV: n=6, *Glb1^-/-^* Placebo: n=6, *Glb1^+/+^* TMEV: n=6, *Glb1^+/+^* Placebo: n=6; 98 dpi: *Glb1^-/-^* TMEV: n=9, *Glb1^-/-^* Placebo: n=5, *Glb1^+/+^* TMEV: n=10, *Glb1^+/+^* Placebo: n=10.

Next, we investigated the microglia/macrophage activation status in the brain during TMEV infection using immunohistochemistry for CD107b (Mac-3). At 4 dpi, there were no statistically significant differences in the relative CD107b^+^ area between the study groups (**Figure 7A1-A5**). At 7 and 14 dpi, TMEV- and mock-infected *Glb1* ^-/-^ mice displayed a significant increase in CD107b^+^ area compared to TMEV- and mock-infected *Glb1^+/+^* mice, respectively (**Figure 7B1-C5**). At 98 dpi, CD107b^+^ brain area was similarly low in all study groups (**Figure 7D1-D5**). Consequently, G_M1_ lysosomal storage seems to amplify microglia/macrophage activation transiently in the brain during TMEV infection. Moreover, increased microglia/macrophage activation in mock-infected *Glb1* ^-/-^ mice might be related to an unspecific and maybe dysregulated reaction of microglia/macrophages caused by G_M1_ lysosomal storage.

In addition to microglia/macrophage number and activation, the GFAP-positive area was determined to investigate the influence of G_M1_ lysosomal storage and TMEV infection on astrocytes in the brain of C57BL/6 mice. However, there were no differences in the GFAP-positive area between the study groups at any of the investigated time points (**Supplementary Figure 7**). Moreover, APP positive spheroids were not detected in the brain of TMEV- and mock-infected *Glb1^+/+^* and *Glb1* ^-/-^ mice.

### 3.4 No difference in clinical disease between TMEV- and mock-infected *Glb1^-/-^* mice

**Figure 8:**
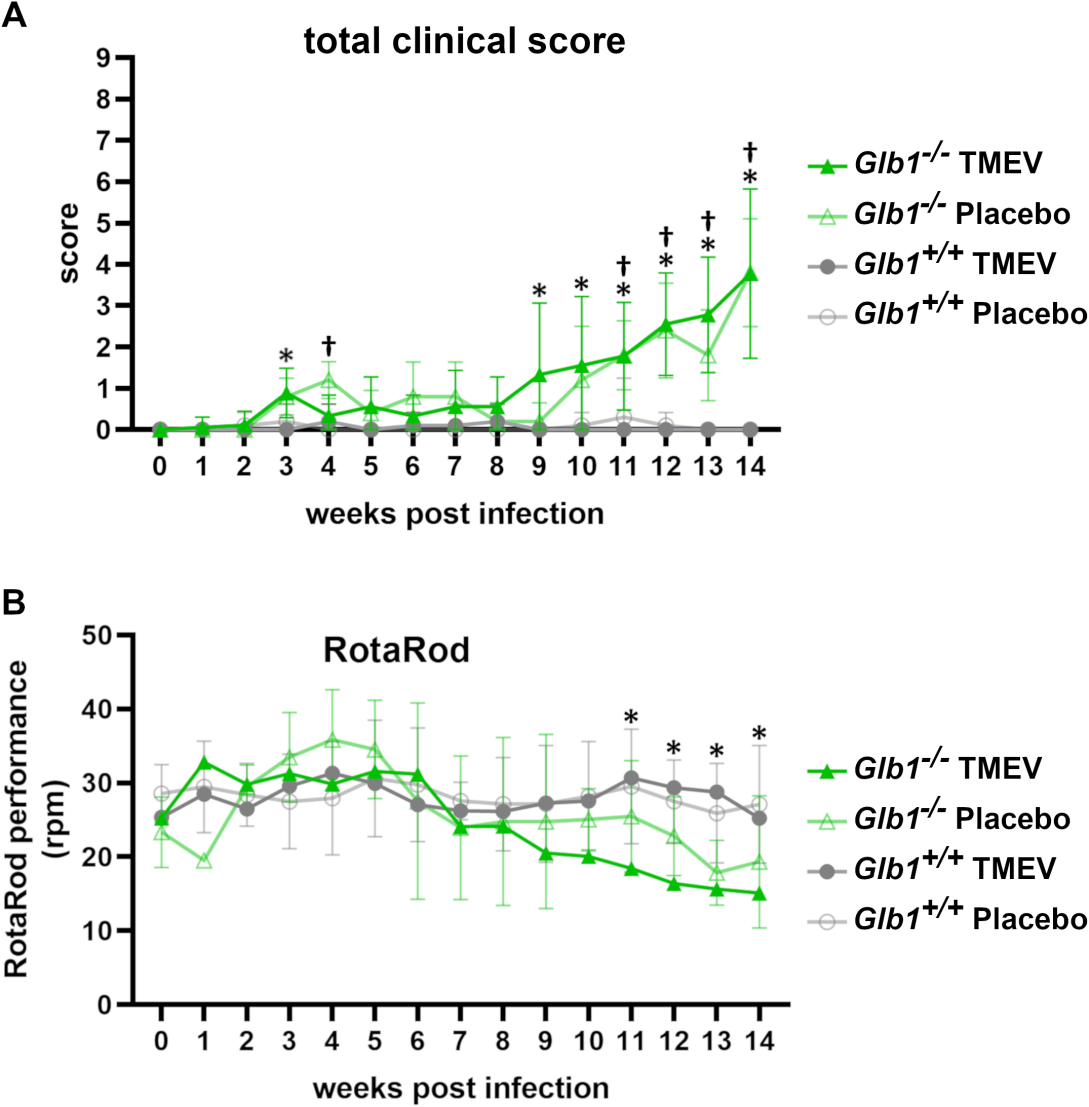
Progression of clinical disease and RotaRod performance of Theiler’s murine encephalomyelitis virus (TMEV)- and mock-infected *Glb1^-/-^* and C57BL/6 wildtype (*Glb1^+/+^*) mice at 0-14 weeks post infection (wpi). **(A)** Total clinical score: Both TMEV-infected *Glb1^-/-^* mice (green line with filled triangles) and mock-infected *Glb1^-/-^* mice (green line with empty triangles) displayed an increase in clinical score compared to *Glb1^+/+^* mice, at 3 and 4 weeks, respectively and starting at 9 and 11 weeks of age, respectively (p<0.05: * TMEV-infected *Glb1^-/-^*, † mock-infected *Glb1^-/-^*). In contrast, clinical scores of TMEV-infected *Glb1^+/+^* mice (grey line with filled-out dots) and mock-infected *Glb1^+/+^* mice (grey line with empty dots) remained at baseline levels throughout the course of the experiment. Progression curves with means and standard deviations. **(B)** Rotarod performance: TMEV-infected *Glb1^-/-^* mice (green line with filled triangles) displayed a progressive decrease in RotaRod performance starting at 11 weeks of age (* p<0.05), whereas a steady RotaRod performance was observed in both TMEV-infected *Glb1^+/+^* mice (grey line with filled-out dots) and mock-infected *Glb1^+/+^* mice (grey line with empty dots) throughout the course of the experiment. Medians with standard deviations. *Glb1^-/-^* TMEV: n=29, *Glb1^-/-^* Placebo: n=25, *Glb1^+/+^* TMEV: n=32, *Glb1^+/+^* Placebo: n=32.

*Glb1* ^-/-^ mice developed clinical signs of G_M1_ LSD at an age of 14-15 weeks (9-10 weeks post infection), which were not altered by TMEV infection (**Figure 8A**). Similarly, there was no difference in RotaRod performance between TMEV- and mock-infected *Glb1* ^-/-^ mice (**Figure 8B**). This finding suggests that while *Glb1^-/-^* mice show clinical signs due to G_M1_ gangliosidosis, they are clinically unaffected by TMEV infection.

In summary, we observed a delayed induction of the immune response in *Glb1^-/-^* compared to *Glb1^+/+^* mice during TMEV infection. However, T cell infiltration and activation as well as microglia/macrophage activation were prolonged in *Glb1^-/-^* mice upon TMEV infection, which may suggest that G_M1_ gangliosidosis leads to a dysregulated immune response during infection. However, TMEV elimination was not substantially impaired in *Glb1^-/-^* mice, which were only affected by G_M1_-associated pathology at the end of the experimental run.

## 4 Discussion

The aim of the study was to discern whether G_M1_ gangliosidosis affects the immune response against neurotropic virus infection, thereby impacting the course of disease. Therefore, clinical disease progression, cerebral virus load and cerebral immune cell composition and -activation were measured in TMEV-infected *Glb1^+/+^* and *Glb1^-/-^* mice. Both *Glb1^+/+^* and *Glb1^-/-^* mice were able to clear the virus from their CNS and displayed no significant difference in their clinical score during the early phases of the experiment. Differences in clinical score between *Glb1^-/-^* and *Glb1^+/+^* mice towards the end of the experiment are likely caused by clinical alterations related to G_M1_ gangliosidosis, as this is consistent with the established onset of clinical symptoms in this mouse model (21). However, TMEV-infected *Glb1^-/-^* mice displayed a delayed expansion of microglia/macrophages and infiltration of T cells and macrophages into the brain, thus allowing accelerated virus replication in the early phase of the disease. In contrast, mock-infected *Glb1^-/-^* mice showed higher cerebral T cell numbers and increased microglia/macrophage activation compared to mock-infected *Glb1^+/+^* mice. These observations could be explained by a decrease in antigen-specific immunoreactivity with a concurrent increase in unspecific microglia/macrophage reactivity.

While alterations in the immune response are only incompletely investigated in G_M1_ gangliosidosis, immunological alterations, both in the form of immunosuppression and immunohyperreactivity/autoimmunity, are a hallmark of most LSDs (25, 26). In general, LSDs can affect the immune system either directly by the perturbations in the biological functions of the undegraded and accumulated endogenous macromolecules or indirectly by the effects of lysosomal dysfunction due to their excessive overload (26). Gangliosides are included in cellular membranes and concentrate in lipid rafts that are dynamic assemblies of cholesterol and sphingolipids associated with various proteins including glycosylphosphatidylinositol (GPI)-anchored proteins as well as immunoglobulin E (IgE), T cell antigen and growth factor receptors (61, 62). Raft-dependent signalling processes activate different tyrosine and serine/threonine kinases and thereby Src kinase, PI3 kinase/AKT-Nrf2, Ras-ERK/MAPK, and Hedgehog signaling pathways (62–64). Gangliosides also serve as a possible receptor structure for toxins, bacteria, and viruses (65, 66). G_M1_ oligosaccharide is abundantly found in neurons and microglia and involved in cell adhesion, proliferation, differentiation, and recognition, apoptosis, and regulation of calcium homeostasis mediating neurotrophic and neuroprotective functions (65, 67–73). Moreover, G_M1_ administration can reduce oxidative stress and suppress the production of the pro-inflammatory cytokines IL-1β, IL-6 and TNF in serum and brain tissues (63). Consequently, disturbed degradation of G_M1_ molecules in the present *Glb1^-/-^* mice likely affects the cellular metabolism, activation status, and cytokine production of neurons and microglia due to their participation in Raft-dependent signalling processes.

Immunological alterations can also be brought about by impaired lysosomal, lysophagosomal and autophagosomal function, which can lead to impaired antigen presentation through MHC-I, MHC-II, and CD1d (25, 26). In mouse models for G_M1_- and G_M2_-Gangliosidosis, an increased expression of MHC-II, Fas and TNF-receptor type 1 as well as increased microglial activation and expansion alongside pro-inflammatory cell infiltration were shown in the brain (25, 27). Similarly, extensive microglial activation and CNS infiltration of macrophage-like cell populations, associated with increased astrocytic macrophage inflammatory protein (MIP) 1-α expression starting at the presymptomatic stage of the disease, were observed in human Sandhoff disease patients and *Hexb^-/-^* mice (74). In *Hexb^-/-^* mice, upregulated genes characteristic for microglia/macrophage activation included cathepsin S, Fc receptor, complement components, and MHC-II. A progressive increase in TNF levels was also described in the spinal cord of *Hexb^-/-^* mice (74). In contrast, reduced levels of IL-1β were found in the brain of acid sphingomyelinase knockout mice, a mouse model of the infantile form of Niemann Pick disease (type A), which is likely caused by a blockage of IL-1β release by astrocytes (75, 76). Furthermore, lysosomal storage of gangliosides were shown to inhibit lipid loading of CD1d molecules in the late endosome/lysosome, leading to reduced numbers of invariant natural killer T cells in several mouse models for LSD, which further caused alterations in cytokine production (77). It has also been hypothesized, that anti-ganglioside autoimmunity, which is often seen in gangliosidoses, is triggered by the impaired degradation of gangliosides on neuronal cell surfaces, leading to autoantibody production and increased microglial activation through the *Fc* receptor common *γ*-chain (26, 28).

In the present study, increased microglia/macrophage activation was demonstrated in both TMEV- and mock-infected *Glb1^-/-^* mice. Though only minor changes were observed in cerebral microglia/macrophage expansion, this increase in microglia/macrophage activation before the onset of clinical disease might also be the result of an increased cytokine/chemokine expression such as MIP1-α similar to *Hexb* ^-/-^ mice. Microglial activation can be caused through the recognition of either pathogen-associated molecular patterns (PAMPs), in this case viral protein, or danger-associated molecular patterns (DAMPs) released by necrotic and highly stressed cells via Toll-like receptor (TLR)-2 and -4 (78, 79). Further, intracytoplasmic dsRNA in microglia, generated during TMEV replication, can be detected by TLR-3 and retinoic acid-inducible gene-1 like receptors (RLRs) especially melanoma differentiation-associated gene 5 (MDA5) (79, 80). These so-called pattern recognition receptors (PRRs) signal via the adapter proteins Toll/IL-1 receptor (TIR) domain-containing adapter-inducing IFN-β (TRIF) and IFN-β-promoter stimulator 1 (IPS-1, alias MAVS or CARDIF) and the transcription factors IRF3/IRF7 and NF-κB resulting in microglia activation and interferon production (79). Hippocampal neurons contained a major part of TMEV antigen in the present study but a lower percentage of microglial cells was most likely infected as well (37). In *Glb1* ^-/-^ mice, LSD may contribute to decreased stability and survivability of cells and stress thereby increasing the release of DAMPs to be recognised by TLR-2 and -4 and RLRs, leading to increased microglial activation. Additionally, *Glb1* ^-/-^ mice displayed a delay in their T cell reactivity and IFN-γ production possibly related to alterations in antigen presentation via MHC-I and MHC-II due to lysosomal dysfunction.

In conclusion, the present results indicate that G_M1_ gangliosidosis is associated with changes in microglia/macrophage activation as well as impairment of the antigen specific immune response, which are likely caused by disturbed antigen presentation and/or T cell reactivity with manifestation prior to the onset of clinical LSD. These alterations in the innate and adaptive immune responses could explain the delayed elimination of TMEV from the CNS. Our findings may also have implications for other neurotropic virus infections. Therefore, future studies should investigate and compare the pathogenesis of other viral CNS infections in LSDs to understand the impact of these hereditary diseases on the antiviral immune response during such infections more in-depth.

## Data Availability

The raw data supporting the conclusions of this article will be made available by the authors, without undue reservation.
