# Corrigendum: Infection of a β-galactosidase-deficient mouse strain with Theiler’s murine encephalomyelitis virus reveals limited immunological dysregulations in this lysosomal storage disease

**DOI:** 10.3389/fimmu.2025.1610299

**Published:** 2025-04-25

**Authors:** Rouven Wannemacher, Felix Stegmann, Deborah Eikelberg, Melanie Bühler, Dandan Li, Sayali Kalidas Kohale, Thanaporn Asawapattanakul, Tim Ebbecke, Marie-Kristin Raulf, Wolfgang Baumgärtner, Bernd Lepenies, Ingo Gerhauser

**Affiliations:** ^1^ Department of Pathology, University of Veterinary Medicine Hannover, Foundation, Hannover, Germany; ^2^ Center for Systems Neuroscience Hannover, Hannover, Germany; ^3^ Institute for Immunology, University of Veterinary Medicine Hannover, Foundation, Hannover, Germany; ^4^ Research Center for Emerging Infections and Zoonoses (RIZ), University of Veterinary Medicine Hannover, Foundation, Hannover, Germany; ^5^ Institute for Parasitology, University of Veterinary Medicine Hannover, Foundation, Hannover, Germany; ^6^ Chair of Biochemistry and Chemistry, Veterinary Faculty, Ludwig-Maximilians-Universität München, Munich, Germany

**Keywords:** β-galactosidase deficiency, brain, GM1 gangliosidosis, microglia activation, T cell activation, Theiler’s murine encephalomyelitis virus

In the published article, there was an error in the **Funding** statement. Two funding sources (“Royal Thai government” and “Brigitte und Prof. Dr. Reiner Müller-Peddinghaus Foundation” are missing. The correct **Funding** statement appears below.

“The author(s) declare that financial support was received for the research and/or publication of this article. DL was funded by the China Scholarship Council (File No. 201606170128). SKK received a scholarship from the Brigitte und Prof. Dr. Reiner Müller-Peddinghaus Foundation. TA received a scholarship from The Royal Thai Government. We acknowledge financial support by the Open Access Publication Fund of the University of Veterinary Medicine Hannover, Foundation.”

The authors apologize for this error and state that this does not change the scientific conclusions of the article in any way. The original article has been updated.

