# Supplementary material for: Infection of a β-galactosidase-deficient mouse strain with Theiler’s murine encephalomyelitis virus reveals limited immunological dysregulations in this lysosomal storage disease
Source: Front Immunol. 2025 Apr 9;16:1467207. doi: 10.3389/fimmu.2025.1467207 (PMC12014673; doi:10.3389/fimmu.2025.1467207)
Supplement: Supplementary file 1 [file DataSheet1.docx]

Supplementary Material

Infection of a β-galactosidase-deficient mouse strain with Theiler’s murine encephalomyelitis virus reveals limited immunological dysregulations in this lysosomal storage disease

Rouven Wannemacher, Felix Stegmann, Deborah Eikelberg, Melanie Bühler, Dandan Li, Sayali Kalidas Kohale, Thanaporn Asawapattanakul, Tim Ebbecke, Marie-Kristin Raulf, Wolfgang Baumgärtner, Bernd Lepenies, Ingo Gerhauser

**Supplemental Table 1. Clinical and neurological scoring procedure**

| **Criteria** | **Score** | | | | |
| --- | --- | --- | --- | --- | --- |
|  | **0** | **1** | **2** | **3** | **4** |
| Weight | <5% weight loss from highest measured weight | >5% weight loss from highest measured weight | >15% weight loss from highest measured weight | >20% weight loss from highest measured weight | n.a. |
| Posture and appearance | Smooth, shiny fur, normal posture | Shaggy, dull, fur, normal posture | Shaggy, dull fur, slightly hunched back | Shaggy fur, arched back, incontinence | n.a. |
| Activity | Active, curious | Very calm, slightly reduced spontaneous activity, normal induced activity | Apathy, moderately reduced spontaneous activity, slightly reduced induced activity | Stupor, no spontaneous activity, significantly reduced induced activity | n.a. |
| Gait | Normal movement | Mild spinal ataxia, occasional, mild to moderate gait insecurity | Moderate spinal ataxia, regular mild to moderate gait insecurity | Severe spinal ataxia, regular moderate to severe gait insecurity | Paralysis of one or more limbs, lack of pain reflex |
| Parachute test | Extension of fore limbs and abduction (>45°) of hind limbs; continuous knee extension | Mildly reduced reaction (<45°); intermittent knee extension | Reduced reaction; flexion and adduction of hind limbs; slow movements | No reaction; continual flexion and adduction of fore- and hind limbs | n.a. |
| Grid walking test | Mouse does not step into a hole for 30 seconds | Mouse steps into a hole after 21-30 seconds | Mouse steps into a hole after 11-20 seconds | Mouse steps into a hole after 0-10 seconds | n.a. |
| Hang test | Mouse holds on for 30 seconds | Mouse holds on for 21-30 seconds | Mouse holds on for 11-20 seconds | Mouse holds on for 0-10 seconds | n.a. |

Legend: n.a.: Not applicable


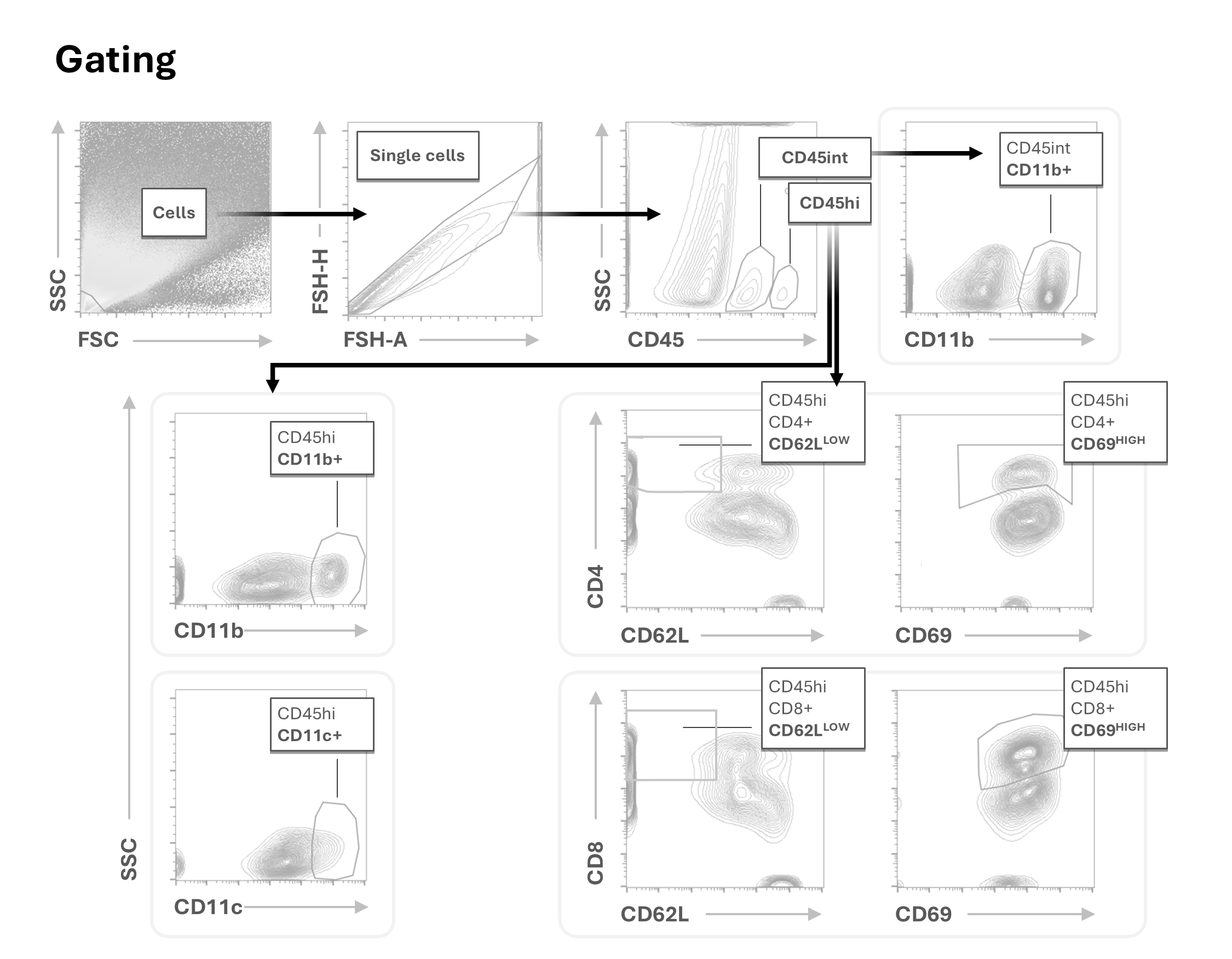


**Supplemental Figure 1. Flow cytometric gating strategy of cells isolated from mouse brains.** Hierarchical gating followed a CD45 staining divided in intermediate (int) and high (hi) signal intensity followed by characterization into CD11b^+^, CD11c^+^, CD4^+^ and CD8^+^ populations. Subsequently, both CD4^+^ and CD8^+^ populations were characterized for activation via selection of CD62L^LOW^ and CD69^HIGH^ events.


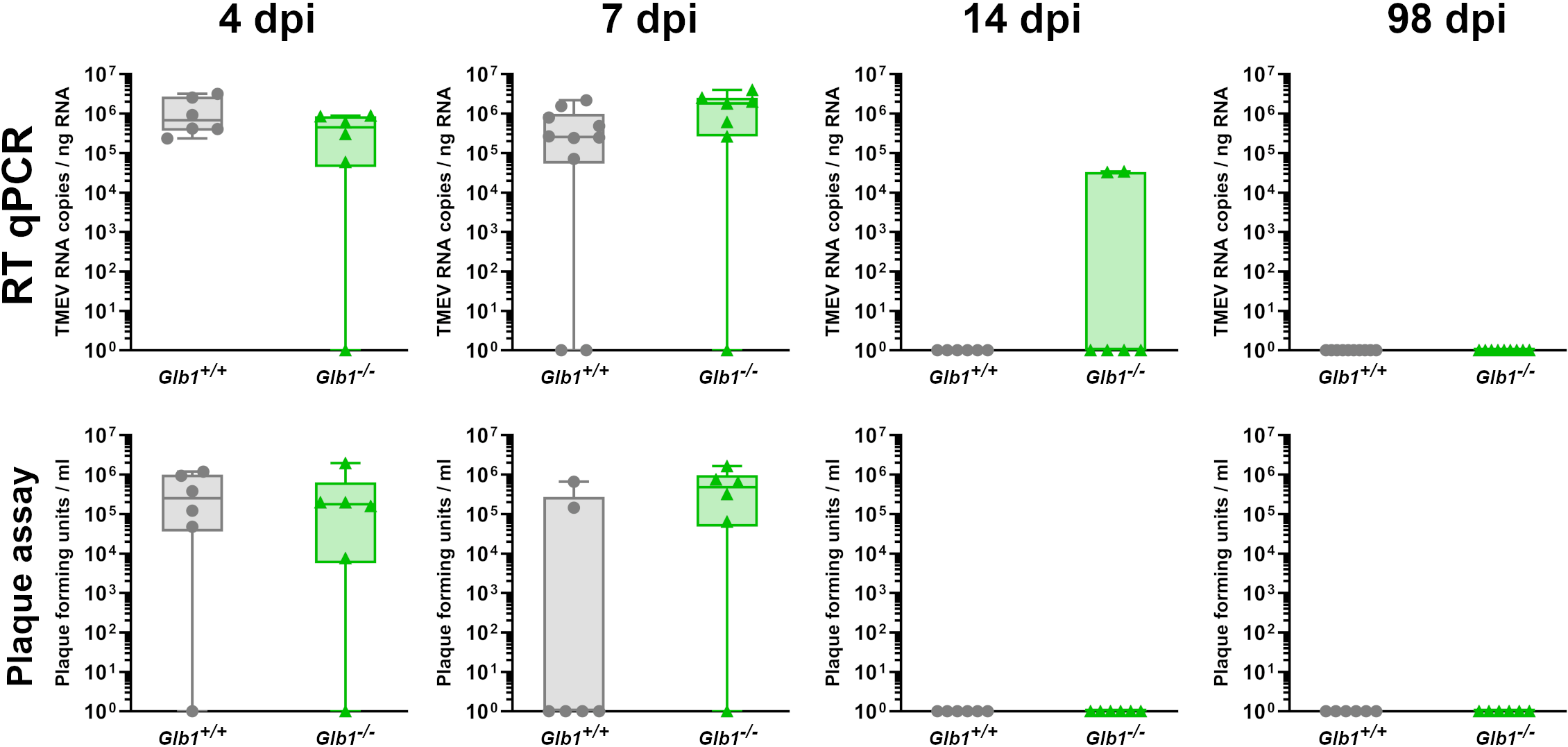


**Supplemental Figure 2. Quantification of infectious Theiler’s murine encephalomyelitis virus (TMEV) via RT-qPCR and plaque assay in the brains of infected *Glb1^-/-^* and C57BL/6 wildtype (*Glb1^+/+^*) mice at 4, 7, 14, and 98 days post infection (dpi).**

At 4 dpi there is a non-significant trend for increased virus load in infected *Glb1^+/+^* mice over *Glb1^‑/‑^* mice.

At 7 dpi *Glb1^-/-^* mice display a non-significant trend for increased virus load over infected *Glb1^+/+^* mice.

Box and whisker plots (min-max) with medians and all data points. *: p<0.05

4 dpi: *Glb1^-/-^* TMEV: n=6, *Glb1^+/+^* TMEV: n=6;

7 dpi: *Glb1^-/-^* TMEV: n=6, *Glb1^+/+^* TMEV: n=6;

14 dpi: *Glb1^-/-^* TMEV: n=6, *Glb1^+/+^* TMEV: n=6;

98 dpi: *Glb1^-/-^* TMEV: n=6, *Glb1^+/+^* TMEV: n=6.


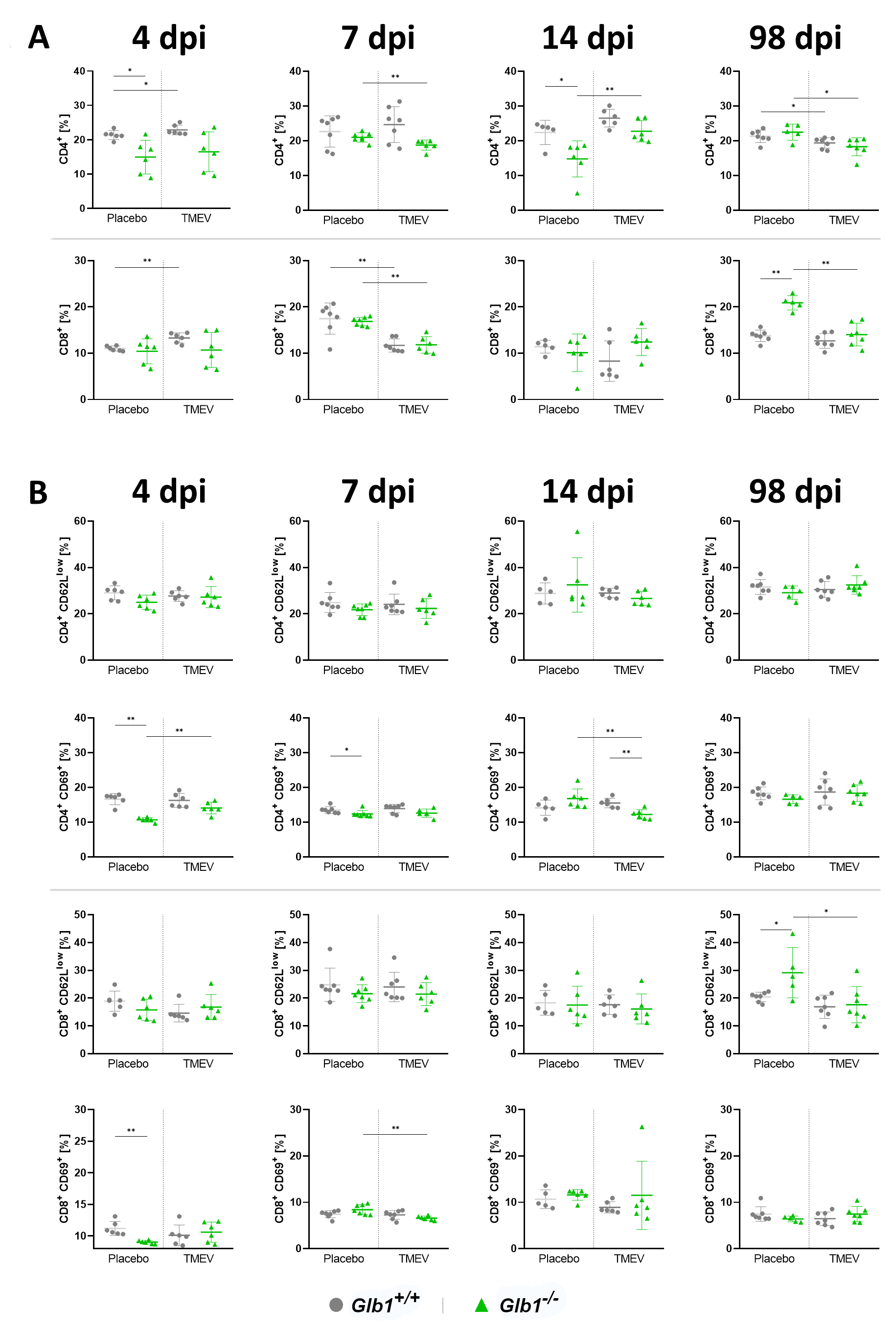


**Supplemental Figure 3 (previous page). Flow cytometry of cells isolated from spleens of Theiler’s murine encephalomyelitis virus (TMEV)- and mock-infected *Glb1*^-/-^ and C57BL/6 wildtype (*Glb1^+/+^*) mice at 4, 7, 14, and 98 days post infection (dpi)**

Both the (A) composition and (B) activation of CD4^+^ and CD8^+^ T cells (rows, from top to bottom) at 4, 7, 14, and 98 dpi (columns, from left to right). Means with all data points. * p < 0.05, ** p < 0.01, with Bonferroni-correction.

4 dpi: *Glb1^-/-^* TMEV: n=6, *Glb1^-/-^* Placebo: n=6, *Glb1^+/+^* TMEV: n=6, *Glb1^+/+^* Placebo: n=6;

7 dpi: *Glb1^-/-^* TMEV: n=8, *Glb1^-/-^* Placebo: n=8, *Glb1^+/+^* TMEV: n=10, *Glb1^+/+^* Placebo: n=10;

14 dpi: *Glb1^-/-^* TMEV: n=6, *Glb1^-/-^* Placebo: n=6, *Glb1^+/+^* TMEV: n=6, *Glb1^+/+^* Placebo: n=6;

98 dpi: *Glb1^-/-^* TMEV: n=9, *Glb1^-/-^* Placebo: n=5, *Glb1^+/+^* TMEV: n=10, *Glb1^+/+^* Placebo: n=10


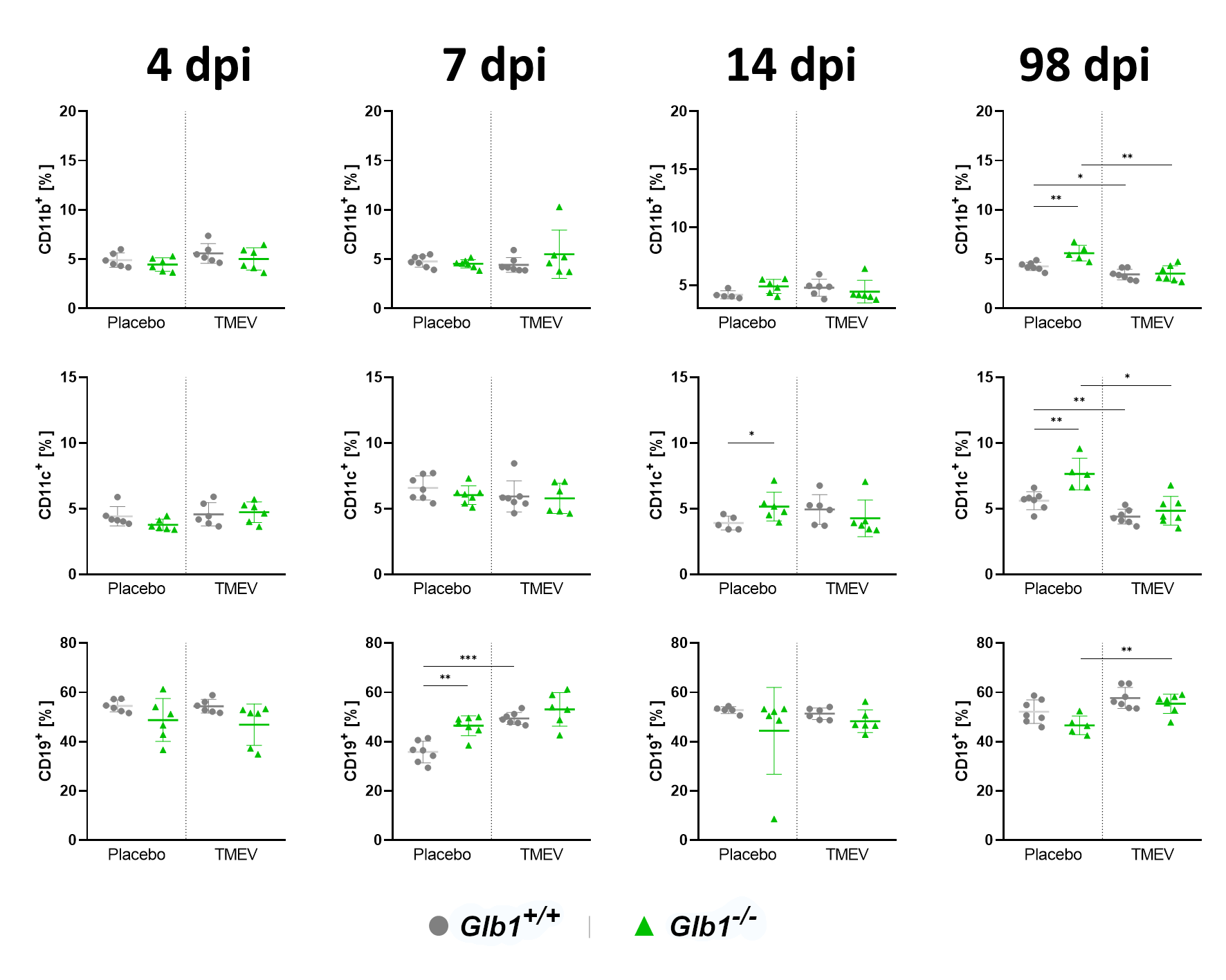
**Supplemental Figure 4. Flow cytometry of cells isolated from spleens of Theiler’s murine encephalomyelitis virus (TMEV)- and mock-infected *Glb1*^-/-^ and C57BL/6 wildtype (*Glb1^+/+^*) mice at 4, 7, 14, and 98 days post infection (dpi)**

Percentage of CD11b^+^, CD11c^+^, and CD19^+^ cells in general (rows, from top to bottom) at 4, 7, 14, and 98 dpi (columns, from left to right). Means with all data points. * p < 0.05, ** p < 0.01, *** p < 0.001 with Bonferroni-correction.

4 dpi: *Glb1^-/-^* TMEV: n=6, *Glb1^-/-^* Placebo: n=6, *Glb1^+/+^* TMEV: n=6, *Glb1^+/+^* Placebo: n=6;

7 dpi: *Glb1^-/-^* TMEV: n=8, *Glb1^-/-^* Placebo: n=8, *Glb1^+/+^* TMEV: n=10, *Glb1^+/+^* Placebo: n=10;

14 dpi: *Glb1^-/-^* TMEV: n=6, *Glb1^-/-^* Placebo: n=6, *Glb1^+/+^* TMEV: n=6, *Glb1^+/+^* Placebo: n=6;

98 dpi: *Glb1^-/-^* TMEV: n=9, *Glb1^-/-^* Placebo: n=5, *Glb1^+/+^* TMEV: n=10, *Glb1^+/+^* Placebo: n=10.


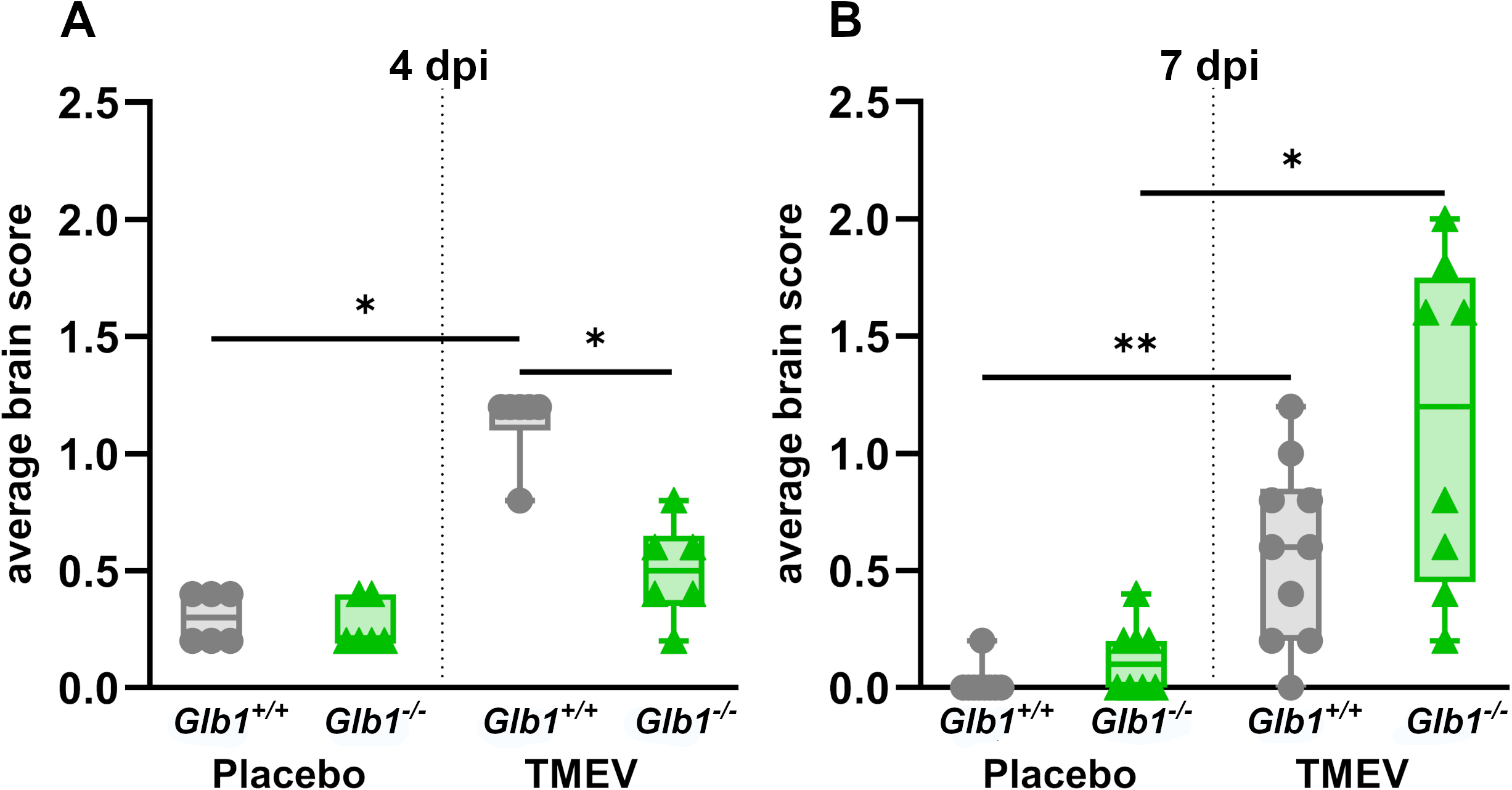


**Supplemental Figure 5. Semiquantitative average score of perivascular infiltration of Iba1-positive macrophages in the brain of Theiler’s murine encephalomyelitis virus (TMEV)- and mock-infected *Glb1^-/-^* and C57BL/6 wildtype (*Glb1^+/+^*) mice at 4 and 7 days post infection (dpi).**

A: At 4 dpi, TMEV-infected *Glb1^+/+^* mice displayed increased perivascular infiltration of macrophages compared to both mock-infected *Glb1^+/+^* mice (p=0.013) and TMEV-infected *Glb1^-/-^* mice (p=0.018).

B: At 7 dpi, TMEV-infected *Glb1^+/+^* mice displayed increased perivascular infiltration of macrophages compared to mock-infected *Glb1^+/+^* mice (p=0.004). Further, TMEV-infected *Glb1^-/-^* mice displayed increased perivascular infiltration of macrophages compared to mock-infected *Glb1^-/-^* mice (p=0.011).

Box and whisker plots (min-max) with medians and all data points. *: p<0.05

4 dpi: *Glb1^-/-^* TMEV: n=6, *Glb1^-/-^* Placebo: n=6, *Glb1^+/+^* TMEV: n=6, *Glb1^+/+^* Placebo: n=6;

7 dpi: *Glb1^-/-^* TMEV: n=8, *Glb1^-/-^* Placebo: n=8, *Glb1^+/+^* TMEV: n=10, *Glb1^+/+^* Placebo: n=10.


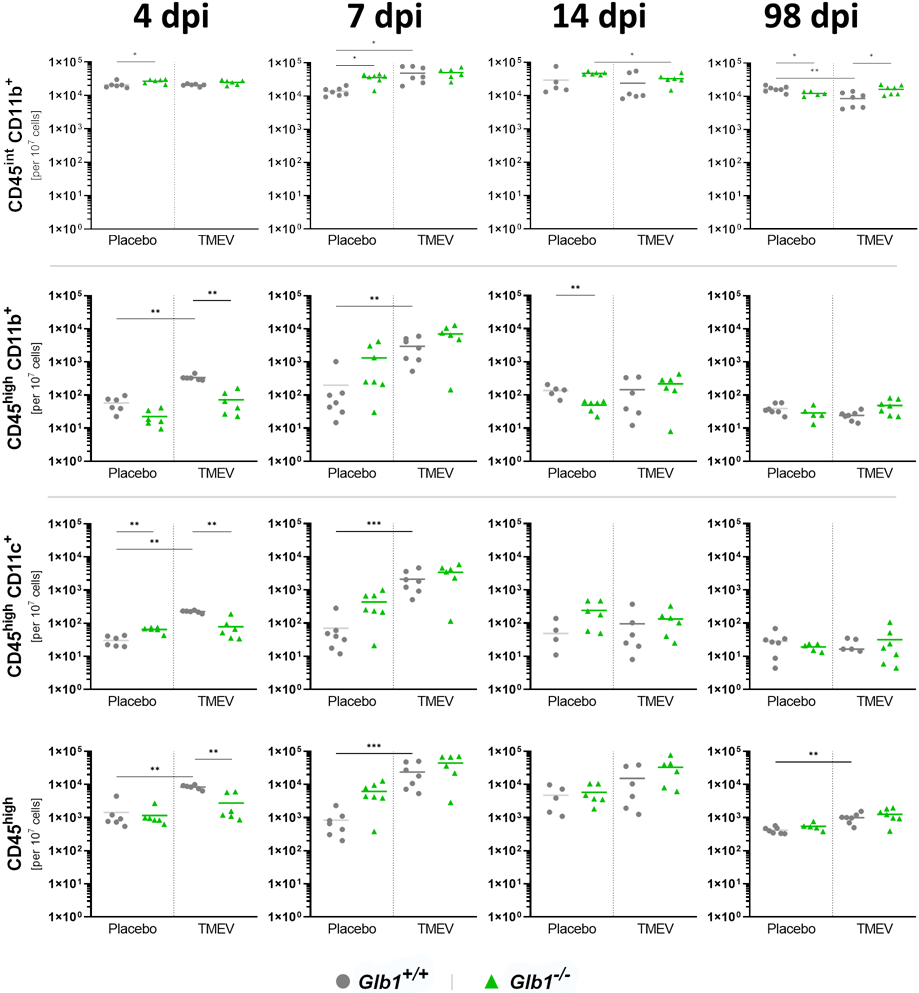


**Supplemental Figure 6. Flow cytometry of cells isolated from brains of Theiler’s murine encephalomyelitis virus (TMEV)- and mock-infected *Glb1*^-/-^ and C57BL/6 wildtype (*Glb1^+/+^*) mice at 4, 7, 14, and 98 days post infection (dpi).**

Total numbers of CD45^int^CD11b^+^ (mostly microglia), CD45^high^CD11b^+^ (mostly macrophages, but also NK cells), CD45^high^CD11c^+^ (mostly dendritic cells) and CD45^high^ cells (leukocytes) in general (rows, from top to bottom) at 4, 7, 14, and 98 dpi (columns, from left to right). Means with all data points. * p < 0.05, ** p < 0.01, *** p < 0.001 with Bonferroni-correction.

4 dpi: *Glb1^-/-^* TMEV: n=6, *Glb1^-/-^* Placebo: n=6, *Glb1^+/+^* TMEV: n=6, *Glb1^+/+^* Placebo: n=6;

7 dpi: *Glb1^-/-^* TMEV: n=8, *Glb1^-/-^* Placebo: n=8, *Glb1^+/+^* TMEV: n=10, *Glb1^+/+^* Placebo: n=10;

14 dpi: *Glb1^-/-^* TMEV: n=6, *Glb1^-/-^* Placebo: n=6, *Glb1^+/+^* TMEV: n=6, *Glb1^+/+^* Placebo: n=6;

98 dpi: *Glb1^-/-^* TMEV: n=9, *Glb1^-/-^* Placebo: n=5, *Glb1^+/+^* TMEV: n=10, *Glb1^+/+^* Placebo: n=10.


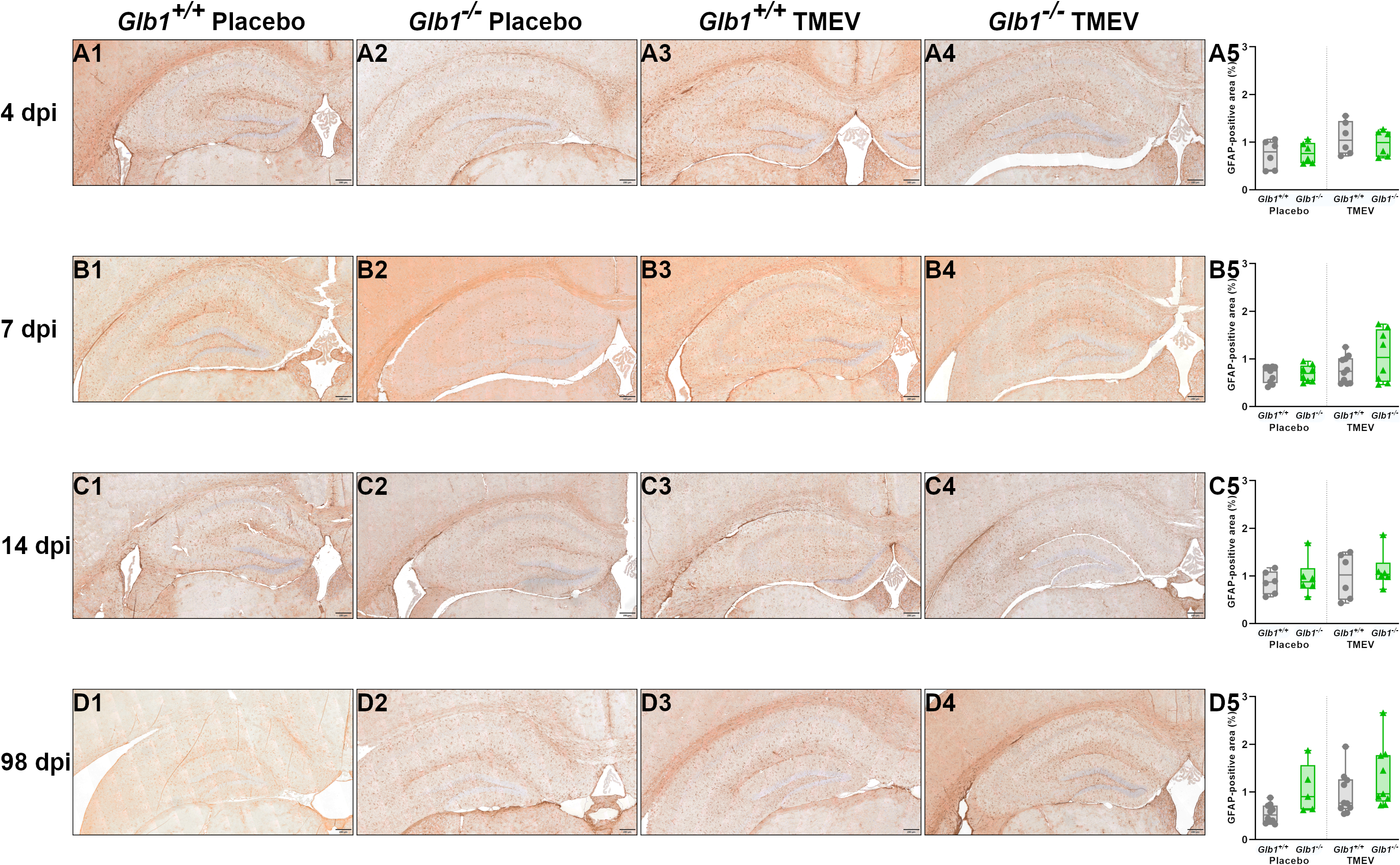


**Supplemental Figure 7. GFAP-positive astrocytes in the brain of Theiler’s murine encephalomyelitis virus (TMEV)- and mock-infected *Glb1^-/-^* and C57BL/6 wildtype (*Glb1^+/+^*) mice at 4, 7, 14, and 98 days post infection (dpi).**

A1-A5, B1-B5, C1-C5, D1-D5: At 4, 7, 14 and 98 dpi, there were no significant differences between the study groups regarding the GFAP-positive area. Box and whisker plots (min-max) with medians and all data points. ABC-DAB immunohistochemistry, glial fibrillary acidic protein (GFAP); Bars (A1-D4) = 500 µm. Images taken from hippocampus

4 dpi: *Glb1^-/-^* TMEV: n=6, *Glb1^-/-^* Placebo: n=6, *Glb1^+/+^* TMEV: n=6, *Glb1^+/+^* Placebo: n=6;

7 dpi: *Glb1^-/-^* TMEV: n=8, *Glb1^-/-^* Placebo: n=8, *Glb1^+/+^* TMEV: n=10, *Glb1^+/+^* Placebo: n=10;

14 dpi: *Glb1^-/-^* TMEV: n=6, *Glb1^-/-^* Placebo: n=6, *Glb1^+/+^* TMEV: n=6, *Glb1^+/+^* Placebo: n=6;

98 dpi: *Glb1^-/-^* TMEV: n=9, *Glb1^-/-^* Placebo: n=5, *Glb1^+/+^* TMEV: n=10, *Glb1^+/+^* Placebo: n=10.
